# Sedentary behavior and health outcomes among older adults: a systematic review

**DOI:** 10.1186/1471-2458-14-333

**Published:** 2014-04-09

**Authors:** Leandro Fornias Machado de Rezende, Juan Pablo Rey-López, Victor Keihan Rodrigues Matsudo, Olinda do Carmo Luiz

**Affiliations:** 1 Department of Preventive Medicine, University of São Paulo School of Medicine, Av. Dr Arnaldo 455 São Paulo, SP Brazil; 2 Center of Studies and Physical Fitness Laboratory from São Caetano do Sul (CELAFISCS), Rua Heloisa Pamplona, 269 Bairro Fundação, São Caetano do Sul, SP Brazil

**Keywords:** Sedentary lifestyle, Sitting time, Television, Risk factors, Aged, Health status, Mortality

## Abstract

**Background:**

In the last decade, sedentary behavior has emerged as a new risk factor for health. The elderly spend most of their awake time in sedentary activities. Despite this high exposure, the impact of this sedentary behavior on the health of this population has not yet been reviewed. We systematically reviewed evidence for associations between sedentary behavior and multiple health outcomes in adults over 60 years of age.

**Methods:**

We searched the Medline, Embase, Web of Science, SPORTDiscus, PsycINFO, CINAHL, LILLACS, and Sedentary Research Database for observational studies published up to May 2013. Additionally, we contacted members of the Sedentary Behaviour Research Network to identify articles that were potentially eligible. After inclusion, the methodological quality of the evidence was assessed in each study.

**Results:**

We included 24 eligible articles in our systematic review, of which only 2 (8%) provided high-quality evidence. Greater sedentary time was related to an increased risk of all-cause mortality in the older adults. Some studies with a moderate quality of evidence indicated a relationship between sedentary behavior and metabolic syndrome, waist circumference, and overweightness/obesity. The findings for other outcomes such as mental health, renal cancer cells, and falls remain insufficient to draw conclusions.

**Conclusion:**

This systematic review supports the relationship between sedentary behavior and mortality in older adults. Additional studies with high methodological quality are still needed to develop informed guidelines for addressing sedentary behavior in older adults.

## Background

Globally, the older adult population has increased substantially, and it is estimated to reach approximately 22% of the world’s population by 2050 [1,2]. The risk of non-communicable diseases and disability increases with age, providing a challenge for health and social care resources [3]. The World Health Organization has created many recommendations for behavior change to reduce the burden of non-communicable diseases and disabilities among the elderly [4]. It is well established that physical activity plays a key role in the prevention of such diseases due to its close relationship with many of the chronic diseases and disabilities that largely affect the elderly, such as cardiovascular disease, cancer, type 2 diabetes, accidental falls, obesity, metabolic syndrome, mental disorders, and musculoskeletal diseases [5,6].

However, in the last decade, sedentary behavior has emerged as a new risk factor for health [7-9]. Sedentary behaviors are characterized by any waking activity that requires an energy expenditure ranging from 1.0 to 1.5 basal metabolic rate and a sitting or reclining posture [10]. Typical sedentary behaviors are television viewing, computer use, and sitting time [10]. Epidemiological studies on different age groups show that a considerable amount of a human’s waking hours are spent in sedentary activities, creating a new public health challenge that must be tackled [11,12]. The scientific study of sedentary behavior has become popular in recent years. In fact, several systematic reviews of sedentary behaviors and health outcomes among children, adolescents, [13-15] and adults [11,16-19] have recently been published. However, insights from these systematic reviews are limited for several reasons. Firstly, some of these systematic reviews did not evaluate the quality of evidence of the reviewed articles [17,16]. Secondly, some reviews included subjects with a wide age range (i.e., >18 years) [16,17]. Therefore, it is currently assumed that the deleterious health effects attributed to sedentary behaviors are similar among both adults (>18 years) and the elderly (>60 years). However, it has been observed that some cardiovascular risk factors (i.e., smoking, obesity, and consumption of alcohol) are less predictive of mortality in a large sample of Scandinavians aged 75 years or older [20].

Furthermore, compared with other age groups, older adults are the most sedentary. Findings from studies in the US and Europe reported that objectively measured sedentary time was higher in those who were older than 50 years [12] and 65 years, [21] respectively. In addition, it has been reported that adults older than 60 years spend approximately 80% of their awake time in sedentary activities which represents 8 to 12 hours per day [12,21,22]. Similarly, Hallal et al. conducted a global assessment in more than 60 countries and found that the elderly had the highest prevalence of reporting a minimum of 4 hours of sitting time daily [23]. Despite this high exposure in the elderly, the health effects of sedentary behavior in this population have not yet been reviewed. Due to this knowledge gap, we systematically reviewed evidence to look for associations between sedentary behavior and multiple health outcomes in adults over 60 years of age.

## Methods

### Identification and selection of the literature

In May 2013, we searched the following databases: Medline, *Excerpta Medica* (EMBASE), Web of Science, SPORTDiscus, PsycINFO, Cumulative Index to Nursing and Allied Health Literature (CINAHL), *Literatura Latino-Americana e do Caribe em Ciências da Saúde* (LILLACS), and the Sedentary Behavior Research Database (SBRD).

The key-words used were as follows: exposure (sedentary behavior, sedentary lifestyles, sitting time, television viewing, driving, screen-time, video game, and computer); primary outcome (mortality, cardiovascular disease, cancer, type 2 diabetes mellitus); and secondary outcome (accidental falls, frail elderly, obesity, metabolic syndrome, mental disorders, musculoskeletal diseases). Further information regarding the search strategy is included in Additional file 1. According to the purpose of this systematic review, observational studies (cross-sectional, case–control, or cohort) involving older adults (all participants >60 years), with no restriction of language or date, were selected in the screening step.

In addition, we contacted the Sedentary Behaviour Research Network (SBRN) members in July 2013 to request references related to sedentary behavior in older adults. The SBRN is a non-profit organization focused on the scientific network of sedentary behavior and health outcomes. Additional information about the SBRN can be found elsewhere (http://www.sedentarybehaviour.org/).

The studies retrieved were imported into the EndNote Web® reference management software to remove any duplicates. Initially, titles and abstracts were screened by two independent reviewers (LFMR and JPRL). Relevant articles were selected for a full read of the article. Disagreements between the two reviewers were settled by a third reviewer. In addition, the reference lists of the relevant articles were reviewed to detect additional articles that were not identified in the previous search strategy.

Studies were excluded if they met the following criteria: 1) Included adults <60 years of age; 2) did not include physical activity as a covariate; or 3) presented only a descriptive analysis of sedentary behavior.

### Data extraction and quality assessment

The data from all of the eligible articles were extracted independently by two reviewers (LFMR and JPRL). The extracted data included the following information: author(s), year, country, age group, number of participants, type of population (general or patient), type of sedentary behavior, type of measurement tool, sedentary definition, adjusted confounders, and outcome (Additional file 2: Table S1).

The quality assessment was performed by two independent reviewers (LFMR, JPRL) and discussed during a consensus meeting. The quality of articles was assessed using the *Grades of Recommendation, Assessment, Development and Evaluation* (GRADE) tool (Table 1). Briefly, the GRADE quality assessment tool begins with the design of the study. Studies with an observational design start with a low quality (2 points). The studies then lose points based on the presence of the following topics: risk of bias (−1 or −2 points), imprecision (−1 or −2 points), inconsistency (−1 or −2 points), and indirectness (surrogate outcome) (−1 or −2 points). However, studies gain points if the following criteria are met: a high magnitude of effect (RR 2–5 or 0.5 – 0.2) (+ 1 or 2 points), adequate confounding adjustment (+1 point), and a dose–response relationship (+1 point). Finally, the quality of the articles is categorized as follows: high (4 points), moderate (3 points), low (2 points), or very low (1 point). Further information about GRADE has been published elsewhere [24].

**Table 1:** Quality of articles assessed using the Grades of Recommendation, Assessment, Development and Evaluation (GRADE)

**Author**	**Year**	**Design**	**Bias**	**Imprecision**	**Indirectness**	**Heterogeneity**	**Magnitude of effect**	**Confounding adjustment**	**Dose–response**	**Rating**
Gardiner et al. [25]	2011	Cross-sectional	−1	−1	−1	−1	0	1	0	1
Lynch et al. [26]	2011	Cross-sectional	−1	−1	−1	0	0	1	0	1
George et al. [27]	2011	Cross-sectional	2	−1	−1	0	0	1	0	3
Stamatakis et al. [28]	2012	Cross-sectional	−1	0	−1	0	0	1	0	1
Frank et al. [29]	2010	Cross-sectional	−2	−1	−1	0	0	1	0	1
Gomez-Cabello et al. [30]	2012	Cross-sectional	−1	−1	−1	0	1	0	0	1
Gomez-Cabello et al. [31]	2012	Cross-sectional	−1	−1	−1	0	0	0	0	1
Buman et al. [32]	2010	Cross-sectional	−2	0	−1	0	0	1	0	1
Hamer et al. [33]	2012	Cross-sectional	−2	0	−1	0	0	1	0	1
Hamer et al. [34]	2012	Cross-sectional	−2	−1	−1	0	0	1	0	1
Bankoski et al. [35]	2011	Cross-sectional	0	−1	−1	0	0	1	0	1
Gao et al. [36]	2007	Cross-sectional	0	−1	−1	0	1	1	1	3
Inoue et al. [37]	2012	Cross-sectional	−2	−1	−1	0	0	1	0	1
Dogra et al. [38]	2012	Cross-sectional	−1	−1	0	0	1	1	0	2
Gennuso et al. [39]	2013	Cross-sectional	0	−1	−1	0	1	1	1	3
Geda et al. [40]	2011	Cross-sectional	−1	0	−1	0	1	1	0	2
Geda et al. [41]	2012	Case–control	−1	−1	−1	0	1	0	0	1
Balboa-Castillo et al. [42]	2011	Prospective Cohort	−2	−1	0	0	1	1	1	2
Campbell et al. [43]	2013	Prospective Cohort	−1	0	0	0	0	1	0	2
Martinez-Gomez et al. [44]	2013	Prospective Cohort	−1	0	0	0	1	1	0	3
Pavey et al. [45]	2012	Prospective Cohort	−1	0	0	0	1	1	1	4
León-Muñoz et al. [46]	2013	Prospective Cohort	−1	0	0	0	1	1	1	4
Verghese et al. [47]	2003	Prospective Cohort	−2	0	0	0	1	1	0	2

## Results

### Search and selection

**Figure 1:**
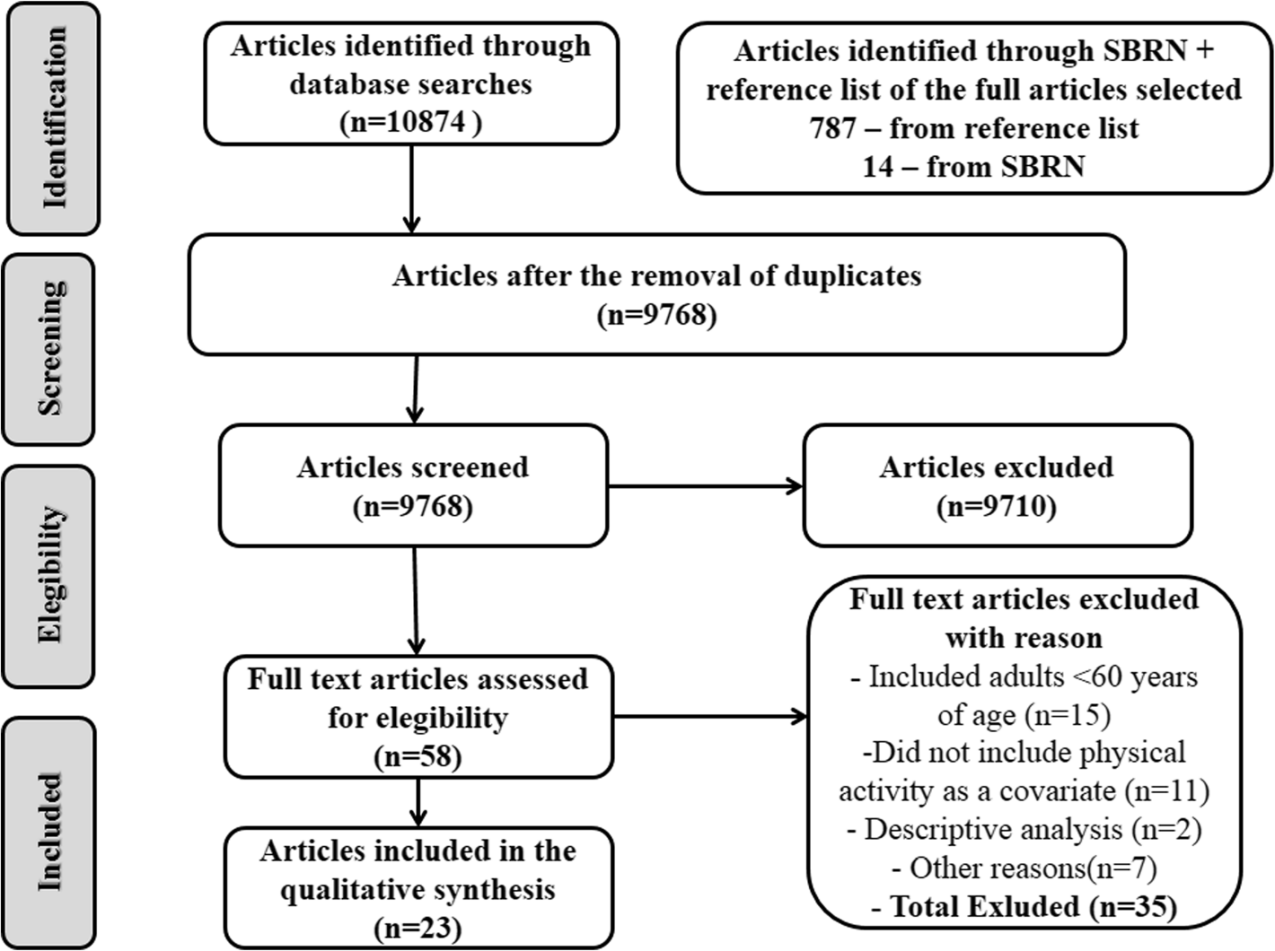
Flowchart outlining study selection.

The search included 10874 potentially relevant articles (1301 from Medline, 5190 from EMBASE, 2803 from Web of Science, 184 from CINAHL, 160 from Lillacs, 154 from SportsDiscus, 936 from PsychInfo, and 146 from Sedentary Behavior Research Database). Fourteen additional records were selected from the articles suggested by the SBRN members (Figure 1).

After removing duplicate records, a total of 9768 articles remained. After screening titles and abstracts, 56 full papers were read in their entirety. In addition, 2 articles were found in the reference list of these full papers (an additional 787 titles were screened). Of the 58 articles, only 23 met the inclusion criteria and were included in the review. The complete list of included and excluded articles is presented in the Additional file 3.

### Methodological quality assessment

Additional file 2: Table S1 presents the quality assessment of the 23 articles included in the review. Of the 23 articles included, 16 (70%) were cross-sectional studies, [25-40] 1 (4%) was a case–control study, [41] and 6 (26%) were prospective cohort studies [42-47]. Concerning quality of the evidence, 12 (52%) were evaluated as very low, [25,26,29-36,38,42] 5 (22%) as low, [39,41,43,44,48] 4 (17%) as moderate, [28,37,40,45] and 2 (9%) as high quality evidence [46,47].

Risk of selection bias was identified in 9 articles (39%), [25,28,29,31-34,37,42,47] and information bias due to self-reported instruments was found in 20 articles (83%) [25-27,31-34,37,38,40-47]. Indirectness (surrogate outcomes) was used in 16 articles (70%), [25-37,39-41] imprecise results were presented in 14 (61%) articles, [25-27,30-32,34-39,41,42] and an inconsistent [25] result among subgroups was found in 1 (4%) article. Most of the articles (n = 20 – 87%) received an additional point for the adjustment of potential confounders [25-29,32-39,41-47]. Eleven (48%) studies gained a point for magnitude of effect [30,36,39,8-42,44-47] and 5 (22%) for considering a dose–response relationship [36,39,42,45,46]. Further details concerning the quality assessment of each article are presented in the Additional file 4.

### Sedentary behavior—health outcomes

#### Mortality

Four prospective cohort studies, [43-46] classified as low, [43] moderate [44] and high quality, [45,46] investigated the relationship between sedentary behavior and mortality (all-cause, cardiovascular, colorectal cancer, other causes).

Martinez-Gomez et al.’s [44] study showed that individuals who spent less than 8 hours sitting/day had a lower risk of all-cause mortality (HR = 0.70, 95% CI: 0.60 to 0.82) when compared with their sedentary peers. In addition, individuals who were physically active and less sedentary (<8 hours/day of sitting) showed a lower risk of all cause-mortality (HR 0.44; 95% CI: 0.36 to 0.52) than those who were inactive and sedentary.

Similarly, Pavey et al. [45] found a dose–response relationship between sitting time and all-cause mortality. Individuals who spent 8–11 hours/day (HR 1.35; 95% CI 1.09 – 1.66) and more than 11 hours/day sitting (HR 1.52; 95% CI 1.17 – 1.98) presented a higher risk of all-cause mortality than those who spent less than 8 hours/day sitting. For each hour/day spent sitting, there was an increase of 3% (HR 1.03; CI 95% 1.01-1.05) in the risk of all-cause mortality. Moreover, the risk of all-cause mortality of individuals who were physically inactive (less than 150 minutes/week) and spent 8–11 or more than 11 hours/day sitting increased by 31% (HR 1.31, 95% CI 1.07 to 1.61) and 47% (HR 1.47, CI 1.15 to 1.93), respectively.

In León-Munoz et al., [46] individuals were classified as consistently sedentary (>median in 2001 and 2003), newly sedentary (<median in 2001 and > median in 2003), formerly sedentary (>median in 2001 and < median in 2003), and consistently nonsedentary (<median in 2001 and 2003). They found that when compared with the consistently sedentary group, subjects newly sedentary (HR 0.91; 95% CI 0.76 - 1.10), formerly sedentary (0.86; 95% CI 0.70 - 1.05), or consistently non-sedentary (0.75; 95% CI 0.62 - 0.90) were protective against all-cause mortality.

Examining a colorectal cancer survivor population, Campbell et al. [43] identified that more than 6 hours per day of pre-diagnosis leisure sitting time, when compared with fewer than 3 hours per day, was associated with a higher risk of all-cause mortality (RR, 1.36; 95% CI, 1.10 to 1.68) and mortality from all other causes (not cardiovascular and colorectal cancer) (RR, 1.48; 95% CI 1.05-2.08). Post-diagnosis (colon cancer) sitting time (>6 hours) was associated with a higher risk of all-cause mortality (RR, 1.27; 95% CI, 0.99 to 1.64) and colorectal cancer-specific mortality (RR, 1.62; 95% CI, 1.07-2.44).

### Metabolic syndrome

Three cross-sectional studies, [25,35,36] classified as very low [25,35] and moderate [36] quality, investigated the relationship between sedentary behavior and metabolic syndrome.

Gardiner et al., [25] showed that individuals who spent most of their time sitting (highest quartile, >3 hours/day) had an increased odds of having metabolic syndrome (men: OR 1.57; CI 95% 1.02 – 2.41 and women: OR 1.56; CI 95% 1.09 – 2.24) when compared with their less sedentary peers (lowest quartile, <1.14 hours/day). In the same study, women who watched more television (highest quartile) increased their risk of metabolic syndrome by 42% (OR 1.42; CI 95% 1.01 – 2.01) when compared with those who watched less television per day (lowest quartile).

In the same sense, Gao et al. [36] showed that individuals in the highest quartile (>7 hours/day) of television watching/day, when compared with those in the lowest quartile (<1 hours/day), had an increased odds (OR 2.2, 95% CI 1.1–4.2) of having metabolic syndrome. In a dose–response relationship, for each hour of television watching/day, there was an increase of 19% in the odds (95% CI 1.1–1.3; p for trend 0.002) of having metabolic syndrome.

Bankoski et al. [35] found that a greater percentage of the time spent in sedentary behavior increased the risk of having metabolic syndrome (only quartile 2 vs. quartile 1, the hours/day of each quartile was not reported; OR 1.58; 95% CI 1.03 - 2.24), whereas breaks in sedentary time throughout the day protected against metabolic syndrome (only quartile 2 vs. quartile 1; OR 1.53; 95% CI 1.05 - 2.23).

### Cardiometabolic biomarkers

Six cross-sectional studies, [25,28,33,34,36,39] classified as of very low [25,28,33,34] and of moderate quality, [36,39] investigated the relationship between sedentary behavior and independent cardiometabolic biomarkers.

### Triglycerides

The likelihood of having high triglycerides was higher in men (Odds Ratio (OR) 1.61; 95% CI 1.01-2.58) and women (OR 1.66; 95% CI 1.14-2.41) who were in the highest quartile of overall sitting time [25]. However, Gao et al. [36] and Gennuso et al. [39] showed that the association between time spent in sedentary behavior and high triglycerides was not statistically significant.

### HDL cholesterol

Gao et al., [36] found that greater time spent viewing television was associated with low HDL cholesterol (2.5; 95% CI 1.0-5.9; p < 0.05). In a study by Gardiner et al., [25] women in the highest quartile of television viewing and men in the highest quartile of overall sitting time presented an OR for low HDL cholesterol of 1.64 (95% CI 1.06-2.54) and 1.78 (95% CI 1.78; 95% CI 1.05-3.02), when compared with the lowest quartile, respectively. However, Gennuso et al. [39] found that the relationship between time spent in sedentary behavior and low HDL cholesterol was not statistically significant (p = 0.29).

### Blood pressure

When compared with the lowest quartile of overall sitting time, the OR for high blood pressure in the third quartile was 1.50 (95% CI 1.03-2.19) [25]. In Gao et al.’s [36] study, greater time viewing television was associated with high blood pressure (2.5; 95% CI 1.0-6.0; p < 0.05). However, Gennuso et al. [39] found that the relationship between time spent in sedentary behavior and systolic blood pressure (p = 0.09) and diastolic blood pressure (p = 0.32) was not statistically significant.

### Plasma Glucose/ Hb1Ac/ Glucose intolerance

Gennuso et al. [39] demonstrated that greater television viewing and sedentary time was associated with higher plasma glucose (p = 0.04). In Gardiner et al.’s [25] study, this relationship was observed only in women (1.45; 95% CI 1.01-2.09; p < 0.05). However, Gao et al. [36] and Stamatakis et al. [28] found that the relationship between television viewing and high fasting glucose and Hb1Ac was not statistically significant.

### Cholesterol ratio and total

Gao et al. [36] demonstrated that greater time in television viewing was associated with a high total-to-HDL cholesterol ratio (OR 2.0; 95% CI 1.1-3.7; p < 0.05). In Stamatakis et al.’s [28] study, self-reported total leisure-time sedentary behavior (β 0.018; 95% CI 0.005-0.032), television viewing (β 0.021; 95% CI 0.002-0.040), and objectively assessed sedentary behavior (β 0.060; 95% CI 0.000-0.121) were associated with cholesterol ratio. However, Gennuso et al. [39] found that the relationship between time spent in sedentary behavior and total cholesterol was not statistically significant (p = 0.50).

### Other cardiometabolic biomarkers

The association between objectively measured sedentary time and pericardial fat [33] and coronary artery calcification [34] was not observed after adjusting for moderate to vigorous physical activity. Gennuso et al. [39] found a positive association between sedentary hours and C-reactive protein (p < 0.01).

### Waist circumference/waist-to-hip ratio/abdominal obesity

Six cross-sectional studies, [25,26,28,30,36,39] classified as being of very low [25,26,28,30] and of moderate [36,39] quality, investigated the relationship between sedentary behavior and waist circumference/waist-to-hip/abdominal obesity.

Gardiner et al. [25] and Gomez-Cabello et al. [30] found that sitting time increased the risk of abdominal obesity by 80% (OR 1.8; 95% CI 1.20-2.64) in both sexes and 81% in women (OR 1.81; 95% CI 1.21-2.70).

In Stamatakis et al.’s [28] study, television time (β 0.416; 95% CI 0.275 - 0.558) and total self-reported leisure-time sedentary behavior (β 0.234; 95% CI 0.129 - 0.339) were positively related to waist circumference. Gao et al. [36] found that greater time in television viewing was associated with high waist-to-hip ratio (3.9; 95% CI 1.08 - 8.4; p < 0.01). Gennuso et al. [39] found that more time spent in objectively measured sedentary behavior was associated with a high waist circumference (p < 0.01). In a colorectal cancer survivor population, [26] sedentary time was not associated with waist circumference.

### Overweight/obesity

Six cross-sectional studies, [28-31,36,37,39] classified as being of very low [28-31,37] and of moderate [36,39] quality, investigated the relationship between sedentary behavior and overweight/obesity.

Gomez-Cabello et al. [30] demonstrated that sitting more than 4 hours/day increased the risk of overweight (OR 1.7; 95% CI 1.06-2.82) and obesity (OR 2.7; 95% CI 1.62-4.66). In a similar study, Gomez-Cabello et al. [31] showed that being seated more than 4 hours/day increased the risk of overweight/obesity (OR 1.42; 95% CI 1.06-1.89) and overfat (1.4 OR; 95% CI 1.14-1.74) in women and the risk of central obesity (OR 1.74; 95% CI 1.21 – 2.49) in men.

Gennuso et al. [39] found that more time spent in objectively measured sedentary behavior was associated with higher BMI (p < 0.01). In Stamatakis et al.’s [28] study, self-reported leisure-time sedentary behavior (β 0.088; 95% CI 0.047 - 0.130) was associated with BMI.

Inoue et al. [37] found that when compared with the reference category (high television(TV)/insufficient moderate to vigorous physical activity (MVPA)), the adjusted ORs (95% CI) of overweight/obesity were 0.93 (95% CI 0.65-1.34) for high TV/sufficient MVPA, 0.58 (95% CI 0.37-0.90) for low TV/insufficient MVPA, and 0.67 (95% CI 0.47-0.97) for low TV/sufficient MVPA. Stamatakis et al. [28] also showed that TV time (β 0.159; 95% CI 0.104-0.215) was positively associated with BMI. However, only Gao et al. [36] found that greater time of television viewing was statistically significantly association with BMI (OR 1.4; 95% 0.7-2.8).

In the only study that evaluated sedentary behavior in transport, Frank et al. [29] showed that ≥1 hour/day sitting in cars was not associated with overweight (0.86 OR. 95% CI 0.51-1.22) or obesity (0.67 OR; 95 CI% 0.41-1.06).

### Mental health (Dementia, mild cognitive impairment, psychological well-being)

Three cross-sectional studies, [32,38,40] one case–control, [41] and two prospective cohort studies, [42,47] classified as very low [32,41] and low quality [38,40,42,47] investigated the relationship between sedentary behavior and mental health (dementia, mild cognitive impairment, and psychological well-being).

In Verghese et al.’s [47] study, individuals who frequently played board games (HR 0.26; 95% CI 0.17-0.57) and read (HR 0.65; 95% CI 0.43-0.97) were less likely to develop dementia.

Buman et al. [32] demonstrated that sedentary time was negatively associated with psychosocial well-being (β -0.03; 95% CI −0.05 - -0.01); p < 0.001. However, Dogra et al. [38] found that 4 hours or more of sedentary behavior per day was not associated with psychologically successful aging.

With regards to mild-cognitive impairment (MCI), reading books (OR 0.67; 95% CI 0.49-0.94), playing board games (OR 0.65; 95% CI 0.47-0.90), craft activities (OR 0.66; 95% CI 0.47-0.93), computer activities (OR 0.50; 95% CI 0.36-0.71), and watching television (OR 0.48; 95% CI 0.27-0.86) were significantly associated with a decreased odds of having MCI [40]. According to Geda et al.’s [41] study, physical exercise and computer use were associated with a decreased likelihood of having MCI (OR 0.36; CI 95% 0.20-0.68).

However, Balboa-Castillo et al. [42] found that the highest quartile of sitting time was negatively associated with mental health (β-5.04; 95% CI −8.87- -1.21); p trend = 0.009.

### Cancer

Only one study, with moderate quality, found no association between time watching television or videos and renal cell carcinoma [27].

## Discussion

To the best of our knowledge, this is the first systematic review to examine the association between sedentary behavior and health outcomes in older people while considering the methodological quality of the reviewed studies. Similar to previous reviews in adults, [16-19,48] the present review shows observational evidence that greater time spent in sedentary activities is related to an increase risk of all-cause mortality in the elderly. However, in these studies, sedentary behavior was measured through self-reported questionnaires (e.g., hours/day of sitting time), which have moderate criterion validity [49]. Studies with a moderate quality of evidence showed a relationship between sedentary behavior and metabolic syndrome, waist circumference, and overweight/obesity. The findings for other outcomes, such as mental health, renal cancer cells, and falls, remain insufficient to draw conclusions.

However, some sedentary activities (e.g., playing board games, craft activities, reading, computer use) were associated with a lower risk of dementia [47]. Thus, future studies should take into account not only the amount of time spent in sedentary behavior but the social and cognitive context in which the activities takes place [50]. To illustrate this point, some studies have shown that video game and computer use, even though classified as sedentary by energy expenditure criteria, may reduce the risk of mental health disorders [51-53].

### Methodological issues

To overcome the limitations of the observational studies available, future longitudinal studies with a high methodological quality are required. Moreover, the primary limitations found in the reviewed articles should be taken into account in future studies (Additional file 4). Based on these limitations, we offer several recommendations for future studies.

### Selection bias

In nearly half of the reviewed articles (10 articles: 42%), the following selection biases were found: a low response rate; the use of independent and non-institutionalized volunteer participants; and an underrepresentation of some population subgroups [25,27-29,31-34,37,41,47].

### Information bias

To date, the use of accelerometers is the most valid and reliable method for evaluating sedentary behavior, although some devices are not able to distinguish sitting and standing posture [54]. In studies of the elderly, 5 days of accelerometer use seems to be sufficient to evaluate the pattern of sedentary behavior [55]. When using accelerometers, future studies should clearly specify the criteria established for non-wear time [56] and use the most accurate sedentary cut-points (150 counts/min) [57] to avoid misclassification. In the current review, all studies used at least 7 days of accelerometry, with a non-wear time criteria of 60 minutes without counts and sedentary cut-points of <100 counts/minute [26,28,32,37,39] or <199 counts/minute [33,34].

Although subjective measurements present a low to moderate reliability, they allow for the evaluation of the contextual dimension of the sedentary activities [49]. In the present review, information bias attributable to self-reported instruments was found in 20 articles (83%) [25-27,29-34,37,38,42,40,41-47]. In this sense, emergent objective methods (e.g., combination of geolocation data combined with acceleration signals in mobile phone) have been developed to obtain a precise and meaningful characteristic of the patterns of sedentary behavior [49].

In addition, most of the studies in this review used different categorization criteria when measuring sedentary behavior [43-46]. This variation in categorization criteria could limit future synthesis of the evidence. We recommend that future studies on the elderly use existing categorizations of sedentary behavior.

### Imprecision

To reduce random error, future epidemiological studies, especially with longitudinal designs, should use an adequate sample size. In the present review, 14 (58%) studies presented imprecise results [25-27,29-31,34-39,41,42].

### Inconsistency

Subgroup and heterogeneity analysis should be performed and reported in future studies to evaluate the consistency of the findings. In the current study, only one article presented the consistency of the findings between subgroups [25].

*Indirectness*: In the current review, indirectness (surrogate outcomes) was present in 17 articles (71%) [25-37,39-41]. Importantly, conclusions obtained with surrogate markers only allow a better understanding of the sedentary behavior physiology. However, researchers should not consider these surrogate markers as synonymous with the endpoint outcomes [58].

Thus, endpoint outcomes (e.g., cardiovascular events, cancer and mortality) should be addressed in future studies.

### Confounding adjustment

The confusion of effects (confounding) is a central issue in epidemiology. Although all of the studies in the present review included some covariates, such as moderate to vigorous physical activity, some residual confounding may be present [59]. Moreover, health status should be measured and included as a covariate, especially in studies of the elderly to avoid confounding [59]. Although most of the articles received better quality scores when they adjusted for potential confounders, [25-29,32-40,42-47] only 3 studies included health status as a covariate [25,45,46]. Future observational studies should include these important covariates in their statistical analysis.

### Dose–response

Although sedentary behavior is a continuous variable, most of the studies categorized it as either an ordinal or a dummy variable. Such categorization could be an important limitation [60,61]. However, if future studies opt to categorize, they should use small intervals with more homogeneous groups that may allow for the observation of a dose–response gradient between sedentary behavior and health outcomes. In the present review, a dose–response was detected in 5 articles [36,39,42,45,46].

## Conclusion

This review confirms previous evidence of the relationship between sedentary behavior and all-cause mortality among adults. Due to the moderate quality of the studies, weak evidence exists regarding other health outcomes (metabolic syndrome, cardiometabolic biomarkers, obesity, and waist circumference). However, of note, some sedentary activities (e.g., playing board games, craft activities, reading, and computer use) had a protective relationship with mental health status (dementia). Future studies should consider the main methodological limitations summarized in this review to improve the current state of the art. Finally, intervention trials that support the observational knowledge are needed to create informed guidelines for sedentary behavior in the elderly.

## Abbreviations

EMBASE: Excerpta Medica; CINAHL: Cumulative Index to Nursing and Allied Health Literature (CINAHL); LILACS: Literatura Latino-Americana e do Caribe em Ciências da Saúde; SBRD: and the Sedentary Behavior Research Database (SBRD); SBRN: Sedentary Behaviour Research Network; GRADE: Grades of Recommendation; Assessment: Development and Evaluation; HR: Hazard Ratio; RR: Relative Risk; OR: Odds Ratio; HDL: High Density Lipoprotein; MVPA: Moderate to vigorous physical activity; TV: Television; BMI: Body mass index; MCI: Mild-cognitive impairment.

## Competing interests

No financial disclosures were reported by the authors of this paper.

## Authors’ contributions

Study Concept and design: LFMR, OCL; Search Strategy: LFMR; Identification and Selection of the Literature: LFMR, JPRL; Data Extraction and Quality Assessment: LFMR, JPRL; Narrative Synthesis: LFMR, JPRL; Drafting of the Manuscript: LFMR, JPRL; Study Supervision: OCL, VKRM. All authors read and approved the final manuscript.

## Authors’ information

LFMR is a master’s student in the Department of Preventive Medicine - University of São Paulo School of Medicine. JPRL is a post-doctoral student in the Department of Preventive Medicine - University of São Paulo School of Medicine. VKRM is a scientific coordinator of CELAFISCS. OCL is a scientific researcher in the Department of Preventive Medicine - University of São Paulo School of Medicine.

## Pre-publication history

The pre-publication history for this paper can be accessed here:

http://www.biomedcentral.com/1471-2458/14/333/prepub

## Supplementary Material

Additional file 1 Search strategy. Click here for file

Additional file 2: Table S1 Characteristics of the included studies. Click here for file

Additional file 3 Included and Excluded articles. Click here for file

Additional file 4 Quality assessment. Click here for file
