# Supplementary material for: Sedentary behavior and health outcomes among older adults: a systematic review
Source: BMC Public Health. 2014 Apr 9;14:333. doi: 10.1186/1471-2458-14-333 (PMC4021060; doi:10.1186/1471-2458-14-333)
Supplement: Additional file 1 — Search strategy. [file 1471-2458-14-333-S1.docx]

**SUPPLEMENTARY FILE 1**

SEARCH STRATEGY

MEDLINE - 09/05/2013

((((((((mortality)) OR (cardiovascular disease)) OR (cancer)) OR (type 2 diabetes mellitus))) OR (((((((accidental falls)) OR (frail elderly)) OR (obesity)) OR (metabolic syndrome)) OR (mental disorders)) OR (musculoskeletal diseases)))) AND (((((((((((((sedentary behaviour)) OR (sedentary behaviours))) OR (((sedentary lifestyle)) OR (sedentary lifestyles))) OR (((television viewing)) OR (tv viewing))) OR (((tv watching)) OR (television watching))) OR (((video game)) OR (video games))) OR (sitting time)) OR (driving)) OR ("screen time")) OR (computer)) AND (((physical activity)) OR (physical inactivity))) Filter: "aged, 80 and over; aged

Medline Search Details

((((("mortality"[Subheading] OR "mortality"[All Fields] OR "mortality"[MeSH Terms]) OR ("cardiovascular diseases"[MeSH Terms] OR ("cardiovascular"[All Fields] AND "diseases"[All Fields]) OR "cardiovascular diseases"[All Fields] OR ("cardiovascular"[All Fields] AND "disease"[All Fields]) OR "cardiovascular disease"[All Fields])) OR ("neoplasms"[MeSH Terms] OR "neoplasms"[All Fields] OR "cancer"[All Fields])) OR ("diabetes mellitus, type 2"[MeSH Terms] OR "type 2 diabetes mellitus"[All Fields])) OR (((((("accidental falls"[MeSH Terms] OR ("accidental"[All Fields] AND "falls"[All Fields]) OR "accidental falls"[All Fields]) OR ("frail elderly"[MeSH Terms] OR ("frail"[All Fields] AND "elderly"[All Fields]) OR "frail elderly"[All Fields])) OR ("obesity"[MeSH Terms] OR "obesity"[All Fields])) OR (metabolic[All Fields] AND ("syndrome"[MeSH Terms] OR "syndrome"[All Fields]))) OR ("mental disorders"[MeSH Terms] OR ("mental"[All Fields] AND "disorders"[All Fields]) OR "mental disorders"[All Fields])) OR ("musculoskeletal diseases"[MeSH Terms] OR ("musculoskeletal"[All Fields] AND "diseases"[All Fields]) OR "musculoskeletal diseases"[All Fields]))) AND (((((((((((sedentary[All Fields] AND ("behaviour"[All Fields] OR "behavior"[MeSH Terms] OR "behavior"[All Fields])) OR (sedentary[All Fields] AND ("behaviours"[All Fields] OR "behavior"[MeSH Terms] OR "behavior"[All Fields] OR "behaviors"[All Fields]))) OR (("sedentary lifestyle"[MeSH Terms] OR ("sedentary"[All Fields] AND "lifestyle"[All Fields]) OR "sedentary lifestyle"[All Fields]) OR ("sedentary lifestyle"[MeSH Terms] OR ("sedentary"[All Fields] AND "lifestyle"[All Fields]) OR "sedentary lifestyle"[All Fields] OR ("sedentary"[All Fields] AND "lifestyles"[All Fields]) OR "sedentary lifestyles"[All Fields]))) OR ((("television"[MeSH Terms] OR "television"[All Fields]) AND viewing[All Fields]) OR (tv[All Fields] AND viewing[All Fields]))) OR ((tv[All Fields] AND watching[All Fields]) OR (("television"[MeSH Terms] OR "television"[All Fields]) AND watching[All Fields]))) OR (("video games"[MeSH Terms] OR ("video"[All Fields] AND "games"[All Fields]) OR "video games"[All Fields] OR ("video"[All Fields] AND "game"[All Fields]) OR "video game"[All Fields]) OR ("video games"[MeSH Terms] OR ("video"[All Fields] AND "games"[All Fields]) OR "video games"[All Fields]))) OR (sitting[All Fields] AND ("time"[MeSH Terms] OR "time"[All Fields]))) OR ("automobile driving"[MeSH Terms] OR ("automobile"[All Fields] AND "driving"[All Fields]) OR "automobile driving"[All Fields] OR "driving"[All Fields])) OR "screen time"[All Fields]) OR ("computers"[MeSH Terms] OR "computers"[All Fields] OR "computer"[All Fields])) AND (("motor activity"[MeSH Terms] OR ("motor"[All Fields] AND "activity"[All Fields]) OR "motor activity"[All Fields] OR ("physical"[All Fields] AND "activity"[All Fields]) OR "physical activity"[All Fields]) OR (("physical examination"[MeSH Terms] OR ("physical"[All Fields] AND "examination"[All Fields]) OR "physical examination"[All Fields] OR "physical"[All Fields]) AND inactivity[All Fields]))) AND ("aged, 80 and over"[MeSH Terms] OR "aged"[MeSH Terms])

EMBASE - 09/05/2013

**#1.26**

**#1.14** AND **#1.25** AND [aged]/lim

**#1.25**

**#1.15** OR **#1.16** OR **#1.17** OR **#1.18** OR **#1.19** OR **#1.20** OR **#1.21** OR **#1.22** OR **#1.23** OR **#1.24**

**#1.24**

**'mortality'**/exp OR **mortality**

**#1.23**

**'musculoskeletal disease'**/exp OR **'musculoskeletal disease'**

**#1.22**

**'mental disease'**/exp OR **'mental disease'**

**#1.21**

**'metabolic syndrome x'**/exp OR **'metabolic syndrome x'**

**#1.20**

**'obesity'**/exp OR **obesity**

**#1.19**

**'falling'**/exp OR **falling**

**#1.18**

**'frail elderly'**/exp OR **'frail elderly'**

**#1.17**

**'non insulin dependent diabetes mellitus'**/exp OR **'non insulin dependent diabetes mellitus'**

**#1.16**

**'neoplasm'**/exp OR **neoplasm**

**#1.15**

**'cardiovascular disease'**/exp OR **'cardiovascular disease'**

**#1.14**

**#1.12** AND **#1.13**

**#1.13**

**#1.2** OR **#1.10** OR **#1.11**

**#1.12**

**#1.1** OR **#1.2** OR **#1.3** OR **#1.4** OR **#1.5** OR **#1.6** OR **#1.7** OR **#1.8** OR **#1.9**

**#1.11**

**'physical inactivity'**/exp OR **'physical inactivity'**

**#1.10**

**'physical activity'**/exp OR **'physical activity'**

**#1.9**

**'video games'**/exp OR **'video games'**

**#1.8**

**'computer'**/exp OR **computer**

**#1.7**

**'screen time'**

**#1.6**

**'car drive'**/exp OR **'car drive'**

**#1.5**

**'television viewing'**/exp OR **'television viewing'**

**#1.4**

**'sitting'**/exp OR **sitting**

**#1.3**

**sedentary** AND (**'behavior'**/exp OR **behavior**)

**#1.2**

**sedentary** AND (**'behaviour'**/exp OR **behaviour**)

**#1.1**

**'sedentary lifestyle'**/exp OR **'sedentary lifestyle**

LILLACS - 09/05/2013

Motor Activity OR Actividad Motora OR Atividade Motora OR Physical Activity OR Sedentary Lifestyle OR Estilo de Vida Sedentario OR Estilo de Vida Sedentário OR "Sitting Time" OR Sentado” OR "Sedentary Time" OR “Tempo Sentado” OR Tiempo Sentado” OR Television OR Televisión OR Televisão OR Automobile Driving OR Conducción de Automóvil OR Condução de Veículo OR Videogame OR Video Game OR Computer OR Computador; Filter: Aged

WEB OF SCIENCE - 09/05/2013

((((("physical inactivity")) OR (physical activity))) AND ((((((((((((((sedentary behavior)) OR (sedentary lifestyles)) OR ("sedentary time")) OR (sitting time)) OR (tv viewing)) OR (television viewing)) OR (tv watching)) OR (television watching)) OR (driving)) OR ("screen based")) OR (video game)) OR (computer)) OR ("screen time"))) AND Topic=(middle aged OR aged OR aged, 80 and over)

SPORTSDISCUS - 09/05/2013

Sedentary behavior OR sedentary behaviour OR sedentary lifestiles OR sedentary time OR “screen time” OR television viewers OR television viewing OR computer OR video games OR driving behavior AND physical activity OR physical inactivity OR sedentary behavior OR sedentary lifestyles OR sedentary behaviour AND older people OR aged OR elderly OR old age AND mortality OR cardiovascular disease OR cancer OR neoplasm OR type 2 diabetes OR diabetes OR obesity OR metabolic syndrome OR falls in old age OR frail elderly OR musculoskeletal system diseases OR mental disorders OR mental health

PSYCHINFO - 09/05/2013

Any Field: ((AnyField:(obesity) OR AnyField:(metabolic syndrome x) OR AnyField:(metabolic syndrome) OR AnyField:(accidental falls) OR AnyField:(frail) OR AnyField:(musculoskeletal disorders) OR AnyField:(mental disease) OR AnyField:(mental disorders)) OR (AnyField:(mortality) OR AnyField:(cardiovascular disease) OR AnyField:(type 2 diabetes mellitus) OR AnyField:(cancer) OR AnyField:(neoplasm))) *AND* Any Field: (((AnyField:(physical activity) OR AnyField:(physical inactivity) OR AnyField:(sedentary) OR AnyField:(sedentary behaviour) OR AnyField:(sedentary behaviour)) AND (AnyField:(sedentary) OR AnyField:(sedentary lifestyle) OR AnyField:(sitting) OR AnyField:(sedentary behavior) OR AnyField:(sedentary behaviour) OR AnyField:(driving behavior) OR AnyField:(television viewing) OR AnyField:("screen time") OR AnyField:(computer) OR AnyField:(video games))) AND (AnyField:(aged) OR AnyField:(older) OR AnyField:(elderly)))

CINAHL - 09/05/2013

Sedentary behavior OR sedentary behaviour OR sedentary lifestiles OR sedentary time OR “screen time” OR television viewers OR television viewing OR computer OR video games OR driving behavior AND physical activity OR physical inactivity OR sedentary behavior OR sedentary lifestyles OR sedentary behaviour AND older people OR aged OR elderly OR old age AND mortality OR cardiovascular disease OR cancer OR neoplasm OR type 2 diabetes OR diabetes OR obesity OR metabolic syndrome OR falls in old age OR frail elderly OR musculoskeletal system diseases OR mental disorders OR mental health
