# Supplementary material for: Sedentary behavior and health outcomes among older adults: a systematic review
Source: BMC Public Health. 2014 Apr 9;14:333. doi: 10.1186/1471-2458-14-333 (PMC4021060; doi:10.1186/1471-2458-14-333)
Supplement: Additional file 2: Table S1 — Characteristics of the included studies. [file 1471-2458-14-333-S2.doc]

| **Table 1: Characteristics of the included studies** | | | | | | | | | | | |
| --- | --- | --- | --- | --- | --- | --- | --- | --- | --- | --- | --- |
| Author | Year | Country | Population | Age | | N | Sedentary Behavior | | | Adjustment | Outcome |
|  |  |  |  | Range | Mean |  | Type | Measurement tool | Definition |  |  |
| Gardiner et al.^25^ | 2011 | Australia | General | >60 | 69 | 1958 | Television viewing and Overall Sitting time | Questionnaire | Quartile | age, education, alcohol consumption, smoking status, diet quality, self-rated health, physical activity, and hormone replacement therapy or estrogen use in women | Metabolic Syndrome, HDL, TG, abdominal obesity, glucose intolerance |
|  |  |  |  |  |  |  |  |  |  |  |  |
| Lynch et al.^26^ | 2011 | USA | Cancer Survirors patiens | 75.4 (7.3) |  | 103 | Sedentary Time | Accelerometer | <100 cpm | age, educational attainment, total energy intake, and moderate-to-vigorous intensity activity | Waist Circumference |
|  |  |  |  |  |  |  |  |  |  |  |  |
|  |  |  |  |  |  |  |  |  |  |  |  |
| George et al.^27^ | 2011 | USA | General |  | 63 | 289,512 | Television viewing and Total sitting | Questionnaire | Television viewing (<1; 1-2; 3-4; 5-6; >7h/day) and Total sitting (<3; 3-4; 5-6; 7-8; >9h/day) | age, race, history of diabetes, smoking, alcohol intake, diet quality, energy intake, age at ﬁrst live birth/parity, and recreational MVPA | Renal Cell Carcinoma |
|  |  |  |  |  |  |  |  |  |  |  |  |
| Stamatakis et al.^28^ | 2012 | UK | General | >60 |  | 2765 (649 with accelerometer) | Objectively-measured sedentary time, Total leisure-time sedentary behaviour, TV viewing time, and non-TV leisure-time sitting | Questionnaire and Accelerometer | Objectively-measured sedentary time (<100 cpm); | Age, sex, employment status, smoking, education, depression (GHQ score) alcohol consumption, fruit and vegetable consumption, cardiovascular medication (diabetes  medication for Hb1Ac), frequency of unhealthy foods consumption, and self-reported MVPA | BMI, waist circumference, cholesterol ratio, and Hb1Ac |
|  |  |  |  |  |  |  |  |  |  |  |  |
| Frank L et al. ^29^ | 2010 | USA | General | >65 |  | 1970 | Time Traveling by car | Questionnaire | >1 hour/day | age, living alone, household income, car, ethnicity, education, gender, walkability, walking trip, and MVPA | Overweight and Obesity |
|  |  |  |  |  |  |  |  |  |  |  |  |
| Gomez-Cabello et al. ^30^ | 2012 | Spain | General | 65-89 | 73.1 (5) | 457 | Sitting Time | Questionnaire | <4, >4 hours/day | Walking hours | Overweight and Obesity |
|  |  |  |  |  |  |  |  |  |  |  |  |
| Gomez-Cabello et al. ^31^ | 2012 | Spain | General | >65 |  | 3136 | Sitting time | Questionnaire | <4, >4 hours/day | Age and Walking time | Central obesity, overweight-obesity, overfat. |
|  |  |  |  |  |  |  |  |  |  |  |  |
| Buman et al. ^32^ | 2010 | USA | General | >65 | 75.4 | 862 | Sedentary Time | Accelerometer | <100 cpm | age, gender, race, education, senior housing resident status, current smoking status, site, marital status, neighborhood-level walkability, neighborhood-level income, and accelerometer wear time, and other activity threshold. | Physical health, psychosocial well-being |
|  |  |  |  |  |  |  |  |  |  |  |  |
| Hamer et al. ^33^ | 2012 | England | General |  | 66 (5.6) | 446 | Sedentary Time | Accelerometer | <199 cpm | Age, sex, registered time, BMI, HDL, LDL, blood pressure, glycated hemoglobin, smoking, statins, and MVPA | Pericardial fat |
|  |  |  |  |  |  |  |  |  |  |  |  |
| Hamer et al. ^34^ | 2012 | Netherlands | General |  | 66 (6) | 443 | Sedentary Time | Accelerometer | <199 com; Tertile | Age, sex, physical activity registered time, employment, statins use, systolic blood pressure, HDL, TG, BMI, Hb A1c | Coronary artery calcification |
|  |  |  |  |  |  |  |  |  |  |  |  |
| Bankoski et al.^35^ | 2011 | USA | General | >60 |  | 1,367 | Sedentary Time | Accelerometer | Sedentary <100 cpm; Duration of sedentary time (hours), % of sedentary time of total wear time, average length of sedentary bout (min), intensity during sedentary time (counts), number of sedetary breaks. | Age, sex, ethnicity, education, alcohol intake, smoking, BMI, diabetes, heart disease and physical activity | Metabolic Syndrome |
|  |  |  |  |  |  |  |  |  |  |  |  |
| Gao et al.^36^ | 2007 | USA (Puerto Rico, Dominican) | General | >60 |  | 455 | Television viewing | Questionnaire | quartile | age, sex, ethnicity, BMI, education, household arrangement, smoking, and current alcohol use, total energy intake, saturated fat intake, polyunsaturated fat intake, trans fat intake, fruit and vegetable intake, and physical activity score, and daily living score | Metabolic Syndrome |
|  |  |  |  |  |  |  |  |  |  |  |  |
| Inoue et al.^37^ | 2012 | Japan | General | 65 - 74 |  | 1806 | Television viewing | Questionnaire | >median TV viewing (high), <mean TV viewing (low) - high TV/insufficient MVPA, high TV/sufficient MVPA, low TV/insufficient MVPA, and low TV/sufficient MVPA | sex, age, education, employment status, city of residence, smoking, drinking, and physical functioning | Overweight and Obesity |
|  |  |  |  |  |  |  |  |  |  |  |  |
| Dogra et al.^38^ | 2012 | Canada | General | >65 |  | 9,478 | Sitting time | Questionnaire | <2, 2-4, 4 hours/day |  | Sucessful aging (Physical, Psychological and Sociological) |
|  |  |  |  |  |  |  |  |  |  |  |  |
| Gennuso et al.^39^ | 2013 | USA | General | >65 |  | 1, 914 | Sedentary Time | Accelerometer | <100 com; quartiles | age, gender, ethinicity, education, income, marital status, alcohol consumption, current smoking status, cardiovascular disease, accelerometer wear time, BMI (except for weight, waist circumference, and BMI), and physical activity | Cardiometabolic risk factors (weight, BMI, waist circumference, systolic BP, diastololic BP, total cholesterol, HDL, HbA1C, CRP, Triglycerides, LDL, plasma glucose) and physical function |
|  |  |  |  |  |  |  |  |  |  |  |  |
| Geda et al^40^ | 2011 | USA | General |  | 70-89 | 1321 | Reading books, reading magazines, craft activities, artistic activities, playing games, computer activities, watching television | Questionnaire | Once a month or less vs any other frequence | age, sex, education, depression, medical comorbidity, and physical exercise | Mild cognitive impairment |
|  |  |  |  |  |  |  |  |  |  |  |  |
| Geda et al^41^ | 2012 | USA | General | 70-93 |  | 926 | Computer activities | Questionnaire | Computer use (yes or no) | age, sex, education, depression, medical comorbidity, and caloric intake. | Mild cognitive impairment |
|  |  |  |  |  |  |  |  |  |  |  |  |
| Balboa-Castillo et al^42^ | 2011 | Spain | General | >62 | 70.3 (5.6) | 1097 | Sitting hours | Questionnaire | Quartile | Age, sex, education, size of municipality of residence, smoking, alcohol consumption, coronary disease, stroke, cancer, chronic obstructive pulmonary disease, diabetes mellitus, arterial hypertension, physical activity, score in SF-36 in the previous measurement | Mental Health |
|  |  |  |  |  |  |  |  |  |  |  |  |
| Campbell et al^43^ | 2013 | USA | Cancer Survirors | >60 |  | 2262 | Leisure-Time Spent Sitting (driving or sitting in a car, sitting on a bus, or sitting on a train; sitting and watching television; and sitting at home reading) | Questionnaire | <3, 3-6, >6 hours/day | age at diagnosis; sex; smoking status; body mass index; red meat intake; Surveillance, Epidemiology, and End Results (SEER) summary stage at diagnosis; recreational physical activity; and education | All-cause mortality, colorectal cancer-specific mortality, cardiovascular disease-specific mortality, mortality from all other causes |
|  |  |  |  |  |  |  |  |  |  |  |  |
| Martinez-Gomez et al^44^ | 2013 | Spain | General | >60 |  | 3,465 | Sitting Time (eating, listening to the radio, watching television, reading, sewing, drinving and so on). | Questionnaire | <8h/day (yes or no) | age, sex, and educational attainment, occupational status, alcohol intake, former drinking, extreme sleep durations, BMI, waist circumference, systolic blood pressure, hypercholesterolemia status, coronary heart disease, stroke, diabetes mellitus, hip fracture, cancer, never smoking or quitting tobacco >15 years very/moderately physically active, healthy diet score > median in the cohort, sleeping 7 to 8h/d, interaction with friends daily. | All-cause mortality |
|  |  |  |  |  |  |  |  |  |  |  |  |
| Pavey et a.^45^ | 2012 | Australia | General | 76-81 |  | 6656 | Sitting time | Questionnaire | 0-4, 4-8, 8-11, >11 hours/day | age, education, marital status, area, smoking, alcohol consumption, BMI, physical activity, number of chronic conditions, self-reported health and assistance with daily tasks | All-cause mortality |
|  |  |  |  |  |  |  |  |  |  |  |  |
| León-Muñoz et al.^46^ | 2013 | Spain | General | >60 |  | 2635 | Sitting time | Questionnaire | Quartile | sex, age, educational level, smoking, alcohol consumption, BMI, physical activity, chronic lung disease, ischemic heart disease, diabetes mellitus, osteomuscular disease, cancer, SF-36, limitations in mobility, and limitations in agility. | All-cause mortality |
|  |  |  |  |  |  |  |  |  |  |  |  |
| Verghese J et al^47^ | 2003 | USA | General | >75 |  | 469 | Playing board games, reading, doing crossword puzzles, and writting | Questionnaire | Rare and Frequent (several times per week) | age, sex, educational level, presence or absence of medical illnesses, score on the Blessed Information–Memory–Concentration test, and participation or nonparticipation in other leisure activities including physical activities. | Dementia |
