# Supplementary material for: Sedentary behavior and health outcomes among older adults: a systematic review
Source: BMC Public Health. 2014 Apr 9;14:333. doi: 10.1186/1471-2458-14-333 (PMC4021060; doi:10.1186/1471-2458-14-333)
Supplement: Additional file 3 — Included and Excluded articles. [file 1471-2458-14-333-S3.doc]

**SUPPLEMENTARY FILE 3**

**Included Articles**

25. Gardiner PA, Healy GN, Eakin EG, Clark BK, Dunstan DW, Shaw JE, Zimmet PZ, and Owen N. Associations between television viewing time and overall sitting time with the metabolic syndrome in older men and women: the Australian Diabetes, Obesity and Lifestyle study. JAGS. 2011;59(5):788-796

26. Lynch BM, Dunstan DW, Winkler E, Healy GN, Eakin E, and Owen N. Objectively assessed physical activity, sedentary time and waist circumference among prostate cancer survivors: findings from the National Health and Nutrition Examination Survey (2003-2006). Eur J Cancer Care. 2011;20:514-519.

27. George SM, Moore SC, Chow WH, Schatzkin A, Hollenbeck AR, and Matthews CE. A Prospective Analysis of Prolonged Sitting Time and Risk of Renal Cell Carcinoma Among 300,000 Older Adults. Ann Epidemiol 2011;21:787–790.

28. Stamatakis E, Davis M, Stathi A, and Hamer M. Associations between multiple indicators of objectively-measured and self-reported sedentary behaviour and cardiometabolic risk in older adults. Prev Med 2012;54:82-87.

29. Frank L, Keer J, Rosenberg D, and King A. Healthy Aging and Where You Live: Community Design Relationships With Physical Activity and Body Weight in Older Americans. J Phys Act Health. 2010;7(Suppl 1):S82-S90

30.Gomez-Cabello A, Vicente-Rodriguez G, Pindado M, Vila S, Casajús JA, Pradas de la Fuente F, and Ara I. Increased risk of Obesity and central obesity in sedentary postmenopausal Women. Nutr Hosp. 2012;27(3):865-870.

31.Gomez-Cabello A, Pedreto-Chamizo R, Olivares PR, Hernández_Perera R, Rodríguez-Marroyo JA, Mata E, Aznar S, Villa JG, Espino-Torón L, Gusi N, González-Gross, Casajús JA, Ara I, and Vicente-Rodríguez G. Sitting time increases the overweight and obesity risk independently of walking time in elderly people from Spain. Maturitas. 2012;73(4):337–343.

32. Buman MP, Hekler EB, Haskell WL, Pruitt L, Conway TL, Cain KL, Sallis JF, Saelens BE, Frank LD, King AC. Objective Light-Intensity Physical Activity Associations With Rated Health in Older Adults. Am J Epidemiol 2010;172:1155–1165

33. Hamer M, Venuraju SM, Urbanova L, Lahiri A, and Steptoe A. Physical activity, sedentary time, and pericardial fat in healthy older adults. Obesity. 2012;20:2113–2117.

34. Hamer M, Venuraju SM, Lahiri A, Rossi A, and Steptoe A. Objectively assessed physical activity, sedentary time, and coronary artery calcification in healthy older adults. Artriolscler Thromb Vasc Biol 2012;32:500-505

35. Bankoski A, Harris TB, McClain JJ, Brychta RJ, Caserotti P, Chen KY, Berrigan D, Troiano RP, and Koster A. Sedentary Activity Associated With Metabolic Syndrome Independent of Physical Activity. Diabetes Care. 2011;34:497–503.

36. Gao X, Nelson ME, Tucker KL. Television viewing is associated with prevalence of metabolic syndrome in hispanic elders. Diabetes Care. 2007;30:694–700.

37. Inoue S, Sugiyama T, Takamiya T, Oka K, Owen N, and Shimomitsu T. Television Viewing Time is Associated with Overweight/Obesity Among Older Adults, Independent of Meeting Physical Activity and Health Guidelines. J Epidemiol 2012;22(1):50-56

38. Dogra S, Stathokostas L. Sedentary Behavior and Physical Activity Are Independent Predictors of Successful Aging in Middle-Aged and Older Adults. J Aging Res. 2012(2012);190654

39. Gennuso KP, Gangnon RE, Matthews CE, Thraen-Borowski KM, and Colbert LH. Sedentary Behavior, Physical Activity, and Markers of Health in Older Adults. Med. Sci. Sports Exerc. 2013;45(8):1493.

40. Geda YE, Topazian HM, Roberts LA, Roberts RO, Knopman DS, Pankratz VS, Christianson TJ, Boeve BF, Tangalos EG, Ivnik RJ, Petersen RC. Engaging in cognitive activities, aging, and mild cognitive impairment: a population based study. J Neuropsychiatry Clin Neurosci. 2011;23(2):149-154.

41. Geda F, Silber TC, Roberts RO, Knopman DS, Christianson TJ, Pankratz VS, Boeve BF, Tangalos EG, and Petersen RC. Computer activities, physical exercise, aging, and mild cognitive impairment: a population-based study. Mayo Clin Proc. 2012;87(5):437-442

42. Balboa-Castillo T, León-Munoz LM, Graciani A, Rodríguez-Artalejo F, Guallar-Castillón P. Longitudinal association of physical activity and sedentary behavior during leisure time with health-related quality of life in community-dwelling older adults. Health and Qual Life Outcomes 2011;27;9:47

43. Campbell PT, Patel AV, Newton CC, Jacobs EJ, and Gapstur SM. Associations of recreational physical activity and leisure time spent sitting with colorectal cancer survival. J Clin Oncol 2013;31(7):876-885

44. Martinez-Gomez D, Guallar-Castillón P, León-Munoz LM, López-Garcia E, and Rodríguez-Artalejo F. Combined impact of traditional and non-traditional health behaviors on mortality: A national prospective cohort study in Spanish older adults. BMC Med 2013;22(11):47

45. Pavey TG, Peeters GG, and Brown WJ. Sitting-time and 9-year all-cause mortality in older women. Br J Sports Med. 2012;0:1–5

46. León-Muñoz LM, Martínez-Gómez D, Balboa-Castillo T, López-García E, Guallar-Castillón P, Rodríguez-Artalejo F. Continued Sedentariness, Change in Sitting Time, and Mortality in Older Adults. Med Sci Sports Exerc. 2013;45(8):1501-1507.

47. Verghese J, Lipton RB, Katz MJ, Hall CB, Derby CA, Kuslansky G, mabrose AF, Sliwinski M, and Buschke H. Leisure Activities and the Risk of Dementia in the Elderly. N Engl J Med. 2003;348(25):2508-2516.

| **Excluded Articles** | **Reason** |
| --- | --- |
| Vallance JK, Eurich D, Marshall AL, Lavallee CM, and Johnson ST. Associations between sitting time and health-related quality of life among older men. Mental Health and Physical Activity. 2013;6(1):46-54 | Age group |
| Lynch BM, Friedenreich CM, Winkler EA, Healy GN, Vallance JK, Eakin EG, Owen N. Associations of objectively assessed physical activity and sedentary time with biomarkers of breast cancer risk in postmenopausal women: findings from NHANES (2003-2006). Breast Cancer Res Treat. 2011;130(1):183-94. | Age group |
| Maeba K and Takenaka K. Factors affecting falls self-efficacy of home-bound elderly people. Nihon Ronen Igakkai Zasshi. 2010;47(4):323-8. | Other health outcome |
| Marcellini F, Giuli C, Papa R, Gagliardi C, Malavolta M, Mocchegiani E. BMI, life-style and psychological conditions in a sample of elderly italian men and women. J Nutr Health Aging. 2010;14(7):515-22. | Descriptive analysis |
| Kim MT, Juon HS, Hill MN, Post W, Kim KB. Cardiovascular disease risk factors in Korean American elderly. West J Nurs Res. 2001;23(3):269-82. | Did not include sedentary behavior |
| Intorre F, Maiani G, Cuzzolaro M, Simpson EE, Catasta G, Ciarapica D, Mauro B, Toti E, Zaccaria M, Coudray C, Corelli S, Palomba L, Polito A. Descriptive data on lifestyle, anthropometric status and mental health in Italian elderly people. J Nutr Health Aging. 2007;11(2):165-74. | Descriptive analysis |
| Lennartsson C, Silverstein M. Does engagement with life enhance survival of elderly people in Sweden? The role of social and leisure activities. J Gerontol B Psychol Sci Soc Sci. 2001;56(6):S335-42. | Did not include physical activity as a covariate |
| Herrera AP, Meeks TW, Dawes SE, Hernandez DM, Thompson WK, Sommerfeld DH, Allison MA, Jeste DV. Emotional and cognitive health correlates of leisure activities in older Latino and Caucasian women. Psychol Health Med. 2011;16(6):661-74 | Did not include physical activity as a covariate |
| Fares D, Barbosa AR, Borgatto AF, Coqueiro Rda S, Fernandes MH. Factors associated with nutritional status of the elderly in two regions of Brazil. Rev Assoc Med Bras. 2012;58(4):434-41. | Did not include physical activity as a covariate |
| McDermott MM, Liu K, Ferrucci L, Tian L, Guralnik JM, Liao Y, Criqui MH. Greater sedentary hours and slower walking speed outside the home predict faster declines in functioning and adverse calf muscle changes in peripheral arterial disease. J Am Coll Cardiol. 2011;57(23):2356-64. | Age group |
| Dodge HH, Kita Y, Takechi H, Hayakawa T, Ganguli M, Ueshima H. Healthy cognitive aging and leisure activities among the oldest old in Japan: Takashima study. J Gerontol A Biol Sci Med Sci. 2008 Nov;63(11):1193-200. | Did not include physical activity as a covariate |
| Hirvensalo M, Rantanen T, Heikkinen E. Mobility difficulties and physical activity as predictors of mortality and loss of independence in the community-living older population. J Am Geriatr Soc. 2000;48(5):493-8. | Did not include sedentary behavior |
| Fox KR, Stathi A, McKenna J, Davis MG. Physical activity and mental well-being in older people participating in the Better Ageing Project. Eur J Appl Physiol. 2007 Jul;100(5):591-602 | Did not include physical activity as a covariate |
| Gregg EW, Cauley JA, Seeley DG, Ensrud KE, Bauer DC. Physical activity and osteoporotic fracture risk in older women. Study of Osteoporotic Fractures Research Group. Ann Intern Med. 1998;129(2):81-8 | Did not include physical activity as a covariate |
| Gierach GL, Chang SC, Brinton LA, Lacey JV Jr, Hollenbeck AR, Schatzkin A, Leitzmann MF.Physical activity, sedentary behavior, and endometrial cancer risk in the NIH-AARP Diet and Health Study. Int J Cancer. 2009;124(9):2139-47 | Age group |
| Moore SA, Hallsworth K, Plötz T, Ford GA, Rochester L, Trenell MI. Physical activity, sedentary behaviour and metabolic control following stroke: a cross-sectional and longitudinal study. PLoS One. 2013;8(1):e55263. | Sedentary behavior as a outcome |
| Jürimäe J, Kums T, Jürimäe T. Plasma adiponectin concentration is associated with the average accelerometer daily steps counts in healthy elderly females. Eur J Appl Physiol. 2010;109(5):823-8. | Did not include physical activity as a covariate |
| Lucas M, Mekary R, Pan A, Mirzaei F, O'Reilly EJ, Willett WC, Koenen K, Okereke OI, Ascherio A. Relation between clinical depression risk and physical activity and time spent watching television in older women: a 10-year prospective follow-up study. Am J Epidemiol. 2011;174(9):1017-27 | Age group |
| Chastin SF, Ferriolli E, Stephens NA, Fearon KC, Greig C. Relationship between sedentary behaviour, physical activity, muscle quality and body composition in healthy older adults. Age Ageing. 2012;41(1):111-4. | Research letter and other health outcome |
| Gill TM, Gahbauer EA, Murphy TE, Han L, Allore HG. Risk factors and precipitants of long-term disability in community mobility: a cohort study of older persons. Ann Intern Med. 2012 Jan 17;156(2):131-40. | Did not include sedentary behavior |
| Allison MA, Jensky NE, Marshall SJ, Bertoni AG, Cushman M. Sedentary behavior and adiposity-associated inflammation: the Multi-Ethnic Study of Atherosclerosis. Am J Prev Med. 2012 Jan;42(1):8-13. | Age group |
| Santos DA, Silva AM, Baptista F, Santos R, Vale S, Mota J, Sardinha LB. Sedentary behavior and physical activity are independently related to functional fitness in older adults. Exp Gerontol. 2012;47(12):908-12. | Other health outcome |
| Zhang M, Xie X, Lee AH, Binns CW. Sedentary behaviours and epithelial ovarian cancer risk. Cancer Causes Control. 2004 Feb;15(1):83-9. | Age group |
| Blair SN, Wei M. Sedentary habits, health, and function in older women and men. Am J Health Promot. 2000 Sep-Oct;15(1):1-8. | Review article |
| Helmink JH, Kremers SP, van Brussel-Visser FN, de Vries NK. Sitting time and Body Mass Index in diabetics and pre-diabetics willing to participate in a lifestyle intervention. Int J Environ Res Public Health. 2011;8(9):3747-58. | Age group |
| Wang S, Lin S, Zhou Y, Wang Z. Social and behavior factors related to aged Chinese women with osteoporosis. Gynecol Endocrinol. 2008;24(10):538-45. | Did not include sedentary behavior |
| Johnson KM, Nelson KM, Bradley KA. Television viewing practices and obesity among women veterans. J Gen Intern Med. 2006;21 Suppl 3:S76-81. | Age group |
| Vance DE, Wadley VG, Ball KK, Roenker DL, Rizzo M. The effects of physical activity and sedentary behavior on cognitive health in older adults. J Aging Phys Act. 2005;13(3):294-313. | Did not include physical activity as a covariate |
| Jenkins KR, Fultz NH. The relationship of older adults' activities and body mass index. J Aging Health. 2008;20(2):217-34. | Age group |
| Kesse-Guyot E, Charreire H, Andreeva VA, Touvier M, Hercberg S, Galan P, Oppert JM. Cross-sectional and longitudinal associations of different sedentary behaviors with cognitive performance in older adults. PLoS One. 2012;7(10):e47831 | Age group |
| Koster A, Caserotti P, Patel KV, Matthews CE, Berrigan D, Van Domelen DR, Brychta RJ, Chen KY, Harris TB. Association of sedentary time with mortality independent of moderate to vigorous physical activity. PLoS One. 2012;7(6):e37696. | Age group |
| Arnardottir NY, Koster A, Van Domelen DR, Brychta RJ, Caserotti P, Eiriksdottir G, Sverrisdottir JE, Launer LJ, Gudnason V, Johannsson E, Harris TB, Chen KY, Sveinsson T. Objective measurements of daily physical activity patterns and sedentary behaviour in older adults: Age, Gene/Environment Susceptibility-Reykjavik Study. Age Ageing. 2013 Mar;42(2):222-9 | Age group |
| Hamer M, Poole L, Messerli-Bürgy N. Television viewing, C-reactive protein, and depressive symptoms in older adults. Brain Behav Immun. 2013;1591(13)00181-5. | Age group |
| Demakakos P, Hamer M, Stamatakis E, Steptoe A. Low-intensity physical activity is associated with reduced risk of incident type 2 diabetes in older adults: evidence from the English Longitudinal Study of Ageing. Diabetologia. 2010 Sep;53(9):1877-85. | Age group |
| Hamer M, Stamatakis E. Screen-based sedentary behavior, physical activity, and muscle strength in the English longitudinal study of ageing. PLoS One. 2013 Jun 3;8(6):e66222 | Age group |
| Gomez-Cabello A, Pedrero-Chamizo R, Olivares PR, Luzardo L, Juez-Bengoechea A, Mata E, Albers U, Aznar S, Villa G, Espino L, Gusi N, Gonzalez-Gross M, Casajus JA, Ara I; EXERNET Study Group. Prevalence of overweight and obesity in non-institutionalized people aged 65 or over from Spain: the elderly EXERNET multi-centre study. Obes Rev. 2011 Aug;12(8):583-92. | Descriptive analysis |
| Gautam R, Saito T, Kai I. Leisure and religious activity participation and mental health: gender analysis of older adults in Nepal. BMC Public Health. 2007;22(7):299. | Did not include physical activity as a covariate |
