# Supplementary material for: Sedentary behavior and health outcomes among older adults: a systematic review
Source: BMC Public Health. 2014 Apr 9;14:333. doi: 10.1186/1471-2458-14-333 (PMC4021060; doi:10.1186/1471-2458-14-333)
Supplement: Additional file 4 — Quality assessment. [file 1471-2458-14-333-S4.docx]

**Gardiner PA, Healy GN, Eakin EG, Clark BK, Dunstan DW, Shaw JE, Zimmet PZ, and Owen N. Associations between television viewing time and overall sitting time with the metabolic syndrome in older men and women: the Australian Diabetes, Obesity and Lifestyle study. JAGS. 2011;59(5):788-796**

**Design: 2.** Cross-sectional

**Risk of Bias:** **-1**. **Selection Bias**: Although the selected sample was not representative of the general population (more educated than non-participants) the association between the exposure and disease should not change in non-participants. **Information bias**: Television viewing time and overall sitting time questionnaires are reliable for use at the population level.

**Imprecision: -1**. In women TV associated with metabolic syndrome (Q1 VS Q4 OR 1.42 (1.01–2.01), lower HDL (Q1 VS Q4 1.64 (1.06–2.54) and glucose intolerance (Q1 VS Q4 1.45 1.01–2.09). Sitting time associated with metabolic syndrome (Q1 VS Q4 - MEN - 1.56 1.09–2.24; WOMEN 1.57 1.02–2.41), high TG (1.61 (Q1 VS Q4 - MEN - 1.01–2.58; WOMEN 1.66 (1.14–2.41), abdominal obesity (Q1 VS Q4 - WOMEN 1.81 (1.21–2.70) and low HDL (Q1 VS Q4 - MEN 1.78; 1.05–3.02)

**Indirectness:** **-1**. “Metabolic syndrome is a clustering of cardiovascular disease risk factors, and its presence is predictive of type 2 diabetes mellitus and all-cause mortality.”

**Inconsistency: -1.** Some of the metabolic biomarkers showed a statistically significance only for one of the sex.

**Magnitude of effect: 0.** In women TV associated with metabolic syndrome (Q1 VS Q4 OR 1.42 (1.01–2.01), lower HDL (Q1 VS Q4 1.64 (1.06–2.54) and glucose intolerance (Q1 VS Q4 1.45 1.01–2.09). Sitting time associated with metabolic syndrome (Q1 VS Q4 - MEN - 1.56 1.09–2.24; WOMEN 1.57 1.02–2.41), high TG (1.61 (Q1 VS Q4 - MEN - 1.01–2.58; WOMEN 1.66 (1.14–2.41), abdominal obesity (Q1 VS Q4 - WOMEN 1.81 (1.21–2.70) and low HDL (Q1 VS Q4 - MEN 1.78; 1.05–3.02)

**Confounding adjustment: +1.** age, education, physical activity, self-rated health, employment, diet, smoking, and alcohol intake and for hormone replacement therapy and strogen use in women.

**Dose-response: 0.** Dose-response relationship could not be detected in this article.

**Lynch BM, Dunstan DW, Winkler E, Healy GN, Eakin E, and Owen N. Objectively assessed physical activity, sedentary time and waist circumference among prostate cancer survivors: findings from the National Health and Nutrition Examination Survey (2003-2006). Eur J Cancer Care. 2011;20:514-519.**

**Design: 2.** Cross-Sectional

**Risk of Bias:** **-1**. **Selection Bias**: No presence. **Information bias**: - **1** Prostate cancer status was self-reported.

**Imprecision: -1**. Sedentary time was not associated with waist circumference (0.678; CI 95% -1.389-2.745; p=0.498). However, with comparatively wide confidence intervals that encompassed some clinically meaningful effect sizes, it is possible that larger studies with more precision could have different findings.

**Indirectness:** **-1**. “Cancer survivors have an increased risk of morbidity and premature mortality related to other chronic diseases, particularly cardiovascular disease.”

**Inconsistency: 0.** Heterogeneity could not be detected in this article.

**Magnitude of effect: 0.** Sedentary time was not associated with waist circumference (β 0.678; CI 95% -1.389-2.745; p=0.498)

**Confounding adjustment: +1.** Age, educational attainment and total energy intake. Model 3: age, educational attainment, total energy intake, and moderate-to-vigorous intensity activity.

**Dose-response: 0.** Dose-response relationship could not be detected in this article.

**George SM, Moore SC, Chow WH, Schatzkin A, Hollenbeck AR, and Matthews CE. A Prospective Analysis of Prolonged Sitting Time and Risk of Renal Cell Carcinoma Among 300,000 Older Adults. Ann Epidemiol 2011;21:787–790.**

**Design: 2.** Prospective Cohort

**Risk of Bias:** **-1**. **Selection Bias**: No presence. **Information bias**: Health related behaviours, BMI and dietary intake by self-reported questionnaires.

**Imprecision: -1**. Watching television or videos for 7 or more hours versus less than 1 hour per day was 0.96 (95%CI: 0.66, 1.38; p trend = 0.707) (Table 1). The HR for those whose total sitting time was 9 or more hours versus less than 3 hours per day was 1.11 (95% CI: 0.87, 1.41; p trend = 0.765)

**Indirectness:** **0**. “Renal Cancer Cell”

**Inconsistency: 0.** Heterogeneity could not be detected in this article.

**Magnitude of effect: 0.** Watching television or videos for 7 or more hours versus less than 1 hour per day was 0.96 (95%CI: 0.66, 1.38; p trend = 0.707) (Table 1). The HR for those whose total sitting time was 9 or more hours versus less than 3 hours per day was 1.11 (95% CI: 0.87, 1.41; p trend = 0.765)

**Confounding adjustment: +1.** age, sex, race, history of diabetes, smoking, alcohol intake, diet quality, energy intake, and recreational moderate-vigorous physical activity.

**Dose-response: 0.** Dose-response relationship could not be detected in this article.

**Stamatakis E, Davis M, Stathi A, and Hamer M. Associations between multiple indicators of objectively-measured and self-reported sedentary behaviour and cardiometabolic risk in older adults. Prev Med 2012;54:82-87.**

**Design: 2.** Cross-Sectional

**Risk of Bias:** **-1**. **Selection Bias**: Had a response rate of 64%. Compared to those excluded, those included in analysis 1 were younger and more likely to have ﬁnished education after the age of 18, drink above the recommended limit, eat more fruit/vegetable, report less SB and more MVPA, and have lower GHQ scores. Compared to those excluded from analysis 2, those included were younger and more likely to be on CVD medication, have lower GHQ scores, and report less SB and more MVPA. Although the sample used in the self-reported analyses is roughly representative of the target population which adds to the ecological validity of the corresponding results, the sample size decreased in model 1 and model2 **Information bias**: no presence.

**Imprecision: 0**. Total self-reported leisure-time SB showed multivariable-adjusted (including for moderate-to-vigorous physical activity) associations with BMI (beta for mean difference in BMI per 30 min/day extra SB: 0.088 kg/m2 , 95% CI 0.047 to 0.130); waist circumference (0.234, 0.129 to 0.339 cm); cholesterol ratio (0.018, 0.005 to 0.032) and diabetes (odds ratio per 30 min/day extra SB: 1.059, 1.030 to 1.089). Similar associations were observed for TV time while non-TV self-reported SB showed associations only with diabetes (1.057, 1.017 to 1.099). Accelerometry SB was associated with waist circumference only (0.633, 0.173 to 1.093.

**Indirectness:** **-1**. “Many of the chronic conditions which older adults suffer including cardiovascular disease, high blood pressure, and type 2 diabetes could be reduced through modiﬁcation of health behaviours.”

**Inconsistency: 0.** Heterogeneity could not be detected in this article.

**Magnitude of effect: 0.** Total self-reported leisure-time SB showed multivariable-adjusted (including for moderate-to-vigorous physical activity) associations with BMI (beta for mean difference in BMI per 30 min/day extra SB: 0.088 kg/m2 , 95% CI 0.047 to 0.130); waist circumference (0.234, 0.129 to 0.339 cm); cholesterol ratio (0.018, 0.005 to 0.032) and diabetes (odds ratio per 30 min/day extra SB: 1.059, 1.030 to 1.089). Similar associations were observed for TV time while non-TV self-reported SB showed associations only with diabetes (1.057, 1.017 to 1.099). Accelerometry SB was associated with waist circumference only (0.633, 0.173 to 1.093.

**Confounding adjustment: +1.** Age, sex, employment status, smoking, education, depression (GHQ score) alcohol consumption, fruit and vegetable consumption, cardiovascular medication (diabetes medication for Hb1Ac), frequency of unhealthy foods consumption, and self-reported MVPA

**Dose-response: 0.** Dose-response relationship could not be detected in this article.

**Campbell PT, Patel AV, Newton CC, Jacobs EJ, and Gapstur SM. Associations of recreational physical activity and leisure time spent sitting with colorectal cancer survival. J Clin Oncol 2013;31(7):876-885**

**Design: 2.** Prospective Cohort

**Risk of Bias:** **-1**. **Selection Bias**: No presence **Information bias**: Although leisure time spent sitting should be reliable and valid (there is no citation of this), self-reported instruments tends to misclassificate the information (non-differentially in this case).

**Imprecision: 0**. Spending 6 or more hours per day of leisure time sitting compared with fewer than 3 hours per day was associated with higher all-cause mortality (prediagnosis sitting time: RR, 1.36; 95% CI, 1.10 to 1.68; postdiagnosis sitting time: RR, 1.27; 95% CI, 0.99 to 1.64).

**Indirectness:** **0**. “Colorectal cancer diagnoses were verified through medical records or linkage with state cancer registries when medical records could not be obtained.

**Inconsistency: 0.** Heterogeneity could not be detected in this article.

**Magnitude of effect: 0.** Spending 6 or more hours per day of leisure time sitting compared with fewer than 3 hours per day was associated with higher all-cause mortality (prediagnosis sitting time: RR, 1.36; 95% CI, 1.10 to 1.68; postdiagnosis sitting time: RR, 1.27; 95% CI, 0.99 to 1.64).

**Confounding adjustment: +1.** age at diagnosis; sex; smoking status; body mass index; red meat intake; Surveillance, Epidemiology, and End Results (SEER) summary stage at diagnosis; recreational physical activity; and education.

**Dose-response: 0.** Dose-response relationship could not be detected in this article.

**Martinez-Gomez D, Guallar-Castillón P, León-Munoz LM, López-Garcia E, and Rodríguez-Artalejo F. Combined impact of traditional and non-traditional health behaviors on mortality: A national prospective cohort study in Spanish older adults. BMC Med 2013;22(11):47**

**Design: 2.** Prospective Cohort

**Risk of Bias:** **-1**. **Selection Bias**: No presence **Information bias**: lifestyle was self-reported, which may have led to recall bias, particularly for assessing physical activity and non-traditional health behaviors).

**Imprecision: 0**. Avoiding excessive sitting had the strongest inverse association (HR = 0.70, 95% CI: 0.60 to 0.82) with mortality. In addition, individuals who were less active/inactive and spent ≥8 h/d seated, those who were very/moderately physically active and spent <8 h/d seated showed a fully-adjusted mortality HR = 0.44 (95% CI: 0.36 to 0.52).

**Indirectness:** **0**. Mortality was the main objective.

**Inconsistency: 0.** Heterogeneity could not be detected in this article.

**Magnitude of effect: +1.** Avoiding excessive sitting had the strongest inverse association (HR = 0.70, 95% CI: 0.60 to 0.82) with mortality. In addition, individuals who were less active/inactive and spent ≥8 h/d seated, those who were very/moderately physically active and spent <8 h/d seated showed a fully-adjusted mortality HR = 0.44 (95% CI: 0.36 to 0.52).

**Confounding adjustment: +1.** age, sex, and educational attainment, occupational status, alcohol intake, former drinking, extreme sleep durations, BMI, waist circumference, systolic blood pressure, hypercholesterolemia status, coronary heart disease, stroke, diabetes mellitus, hip fracture, cancer, never smoking or quitting tobacco >15 years very/moderately physically active, healthy diet score > median in the cohort, sleeping 7 to 8h/d, interaction with friends daily.

**Dose-response: 0.** Dose-response relationship could not be detected in this article.

**Geda F, Silber TC, Roberts RO, Knopman DS, Christianson TJ, Pankratz VS, Boeve BF, Tangalos EG, and Petersen RC. Computer activities, physical exercise, aging, and mild cognitive impairment: a population-based study. Mayo Clin Proc. 2012;87(5):437-442**

**Design: 2.** Case-Control

**Risk of Bias:** **-1**. **Selection Bias**: No information of non-participants. **Information bias**: Another limitation of our study is recall bias, which is an unavoidable drawback of any survey –based study. However, the data on cognitive activities were collected before determination of whether a person had MCI

**Imprecision: -1**. Compared with the reference group (ie, no moderate physical exercise and no computer use), computer use but no exercise showed a protective factor (OR [95% CI], 0.53 [0.27-1.02]; P .058) to mild impairment.

**Indirectness:** -**1**. Mild cognitive impairment (MCI) is an intermediate stage between the cognitive changes of normal aging and dementia

**Inconsistency: 0.** Heterogeneity could not be detected in this article.

**Magnitude of effect: +1.** Compared with the reference group (ie, no moderate physical exercise and no computer use), computer use but no exercise showed a protective factor (OR [95% CI], 0.53 [0.27-1.02]; P .058) to mild impairment.

**Confounding adjustment: 0.** age, sex, education, depression, medical comorbidity, and caloric intake.

**Dose-response: 0.** Dose-response relationship could not be detected in this article.

**Frank L, Keer J, Rosenberg D, and King A. Healthy Aging and Where You Live: Community Design Relationships With Physical Activity and Body Weight in Older Americans. J Phys Act Health. 2010;7(Suppl 1):S82-S90**

**Design: 2.** Cross-Sectional

**Risk of Bias:** **-2**. **Selection Bias**: it was conducted in a region with limited variability in urban form **Information bias**: Although “traveled in a car at least 1 hour a day” vs. “did not travel in a car for more than 1 hour a day” were derived from the travel survey data measurement should be reliable and valid, self-reported instruments tends to misclassificate the information (non-differentially in this case).

**Imprecision: -1**. 1 hour or more spent sitting in car was not associated with overweight (0.86 OR. 95% CI 0.51-1.22) and obesity (0.67 OR; 95 CI% 0.41-1.06) when compared with <1 hour in car.

**Indirectness:** **-1**. “Large numbers of older adults are afflicted by chronic disease related to obesity, including heart disease, hypertension, cancer, diabetes, COPD, and arthritis.”

**Inconsistency: 0.** Heterogeneity not could be detected in this article.

**Magnitude of effect: 0.** 1 hour or more spent sitting in car was not associated with overweight (0.86 OR. 95% CI 0.51-1.22) and obesity (0.67 OR; 95 CI% 0.41-1.06) when compared with <1 hour in car.

**Confounding adjustment: +1.** age, living alone, household income, car, ethnicity, education, gender, walkability, walking trip, and moderate-vigorous physical activity.

**Dose-response: 0.** Dose-response relationship could not be detected in this article

**Gomez-Cabello A, Vicente-Rodriguez G, Pindado M, Vila S, Casajús JA, Pradas de la Fuente F, and Ara I. Increased risk of Obesity and central Obesity in sedentary postmenopausal Women. Nutr Hosp. 2012;27(3):865-870.**

**Design: 2.** Cross-Sectional

**Risk of Bias:** **-1**. **Selection Bias**: Although the participation rate of the study was 87.1%, the sample included non-institutionalized seniors, from urban cities (6 regions). **Information bias**: Although non-physical hobby activities were derived from the travel survey data measurement should be reliable and valid, self-reported instruments tends to misclassificate the information (non-differentially in this case).

**Imprecision: -1**. Sitting increased the risk of overweight (OR 1.7; 95% CI 1.06-2.82), obesity (OR 2.7; 95% CI 1.62-4.66) and abdominal obesity (OR 1.8; 95% CI 1.20-2.64).

**Indirectness:** **-1**. “Teniendo en cuenta que estos cambios en la composición corporal están relacionados con un aumento de problemas de salud, como hipertensión, problemas cardiovasculares, diabetes, artritis, algunos tipos de cáncer y mortalidad prematura”

**Inconsistency: 0.** Heterogeneity not could be detected in this article.

**Magnitude of effect: +1.** Sitting increased the risk of overweight (OR 1.7; 95% CI 1.06-2.82), obesity (OR 2.7; 95% CI 1.62-4.66) and abdominal obesity (OR 1.8; 95% CI 1.20-2.64).

**Confounding adjustment: 0.** Walking hours

**Dose-response: 0.** Dose-response relationship could not be detected in this article.

**Balboa-Castillo T, León-Munoz LM, Graciani A, Rodríguez-Artalejo F, Guallar-Castillón P. Longitudinal association of physical activity and sedentary behavior during leisure time with health-related quality of life in community-dwelling older adults. Health and Qual Life Outcomes 2011;27;9:47**

**Design: 2.** Prospective Cohort

**Risk of Bias:** **-2**. **Selection Bias**: Losses to follow-up could affect the representativeness of our cohort. **Information bias**: Although sitting measurement should be reliable and valid, self-reported instruments tends to misclassificate the information (non-differentially in this case).

**Imprecision: -1**. Lower quartile of sitting time, those in the upper quartile had worse scores on the scales of physical functioning (β -9.21; 95% CI -13.36 to -5.04), physical role (β -11.96; 95% CI -19.33 to -4.59), bodily pain (β -6.58; 95% CI -11.51 to -1.64), vitality (β 5.04; 95% CI -9.21 to -0.88) and social functioning (β 6.36 95% CI -11.17 to -1.56).

**Indirectness:** **0**. Physical activity reduces the risk of numerous diseases, like ischemic heart disease,[1] stroke,[2] diabetes mellitus[3], and cognitive disorders,[4] as well as total mortality.

**Inconsistency: 0.** Heterogeneity was not mesured found.

**Magnitude of effect: +1** Lower quartile of sitting time, those in the upper quartile had worse scores on the scales of physical functioning (β -9.21; 95% CI -13.36 to -5.04), physical role (β -11.96; 95% CI -19.33 to -4.59), bodily pain (β -6.58; 95% CI -11.51 to -1.64), vitality (β 5.04; 95% CI -9.21 to -0.88) and social functioning (β 6.36 95% CI -11.17 to -1.56).

**Confounding adjustment: +1.** Age, sex, education, size of municipality of residence, smoking, alcohol consumption, coronary disease, stroke, cancer, chronic obstructive pulmonary disease, diabetes mellitus, arterial hypertension, physical activity, score in SF-36 in the previous measurement.

**Dose-response: +1.** Lower quartile of sitting time, those in the upper quartile had worse scores on the scales of physical functioning (β -9.21; 95% CI -13.36 to -5.04; p trend<0.0001), physical role (β -11.96; 95% CI -19.33 to -4.59; p trend =0.005), bodily pain (β -6.58; 95% CI -11.51 to -1.64; p trend 0.03 ), vitality (β 5.04; 95% CI -9.21 to -0.88; p trend = 0.01), social functioning (β 6.36 95% CI -11.17 to -1.56; p trend 0.008) and mental health (β-5.04; 95% CI -8.87- -1.21; p trend 0.009)

**Buman MP, Hekler EB, Haskell WL, Pruitt L, Conway TL, Cain KL, Sallis JF, Saelens BE, Frank LD, King AC. Objective Light-Intensity Physical Activity Associations With Rated Health in Older Adults. Am J Epidemiol 2010;172:1155–1165**

**Design: 2.** Cross-Sectional

**Risk of Bias:** **-2**. **Selection Bias**: Although our physical activity estimates seem in line with those from a nationally representative sample, racial minorities and less educated individuals may have been underrepresented, and these groups are likely to report their health more poorly. **Information bias**: study relied on rated health parameters and did not assess objective health indicators

**Imprecision: 0**. Sedentary time was positively related to physical health ( β -0.02; 95% CI -0.00 - -0.03; p< 0.0001) and psychological well-being (β -0.03; 95% CI -0.05 - -0.01; p< 0.001).

**Indirectness:** **-1**. “Objective measures of light physical activity are therefore needed to examine the impact of such activities on health and well-being

**Inconsistency: 0.** Heterogeneity not could be detected in this article.

**Magnitude of effect: 0.** Sedentary time was positively related to physical health ( β -0.02; 95% CI -0.00 - -0.03; p< 0.0001) and psychological well-being (β -0.03; 95% CI -0.05 - -0.01; p< 0.001).

**Confounding adjustment: +1.** age, gender, race, education, senior housing resident status, current smoking status, site, marital status, neighborhood-level walkability, neighborhood-level income, and accelerometer wear time, and other activity threshold.

**Dose-response: 0.** Dose-response relationship could not be detected in this article.

**Hamer M, Venuraju SM, Lahiri A, Rossi A, and Steptoe A. Objectively assessed physical activity, sedentary time, and coronary artery calcification in healthy older adults. Artriolscler Thromb Vasc Biol 2012;32:500-505**

**Design: 2.** Cross-Sectional

**Risk of Bias:** **-2**. **Selection Bias**: The participants included in the present analysis were generally healthier than the overall Whitehall II sample and demonstrated higher activity levels compared with similar aged British cohort. **Information bias**: The accelerometry device used in the present study could not distinguish between sitting and standing.

**Imprecision: -1**. There was no association between sedentary time and presence of detectable CAC (OR 0.93; 95% CI 0.54–1.59)

**Indirectness:** **-1**. CAC measures cannot reliably identify more vulnerable lesions.

**Inconsistency: 0.** Heterogeneity not could be detected in this article.

**Magnitude of effect: 0.** There was no association between sedentary time and presence of detectable CAC (OR 0.93; 95% CI 0.54–1.59)

**Confounding adjustment: +1.** Age, sex, physical activity registered time, employment, statins use, systolic blood pressure, HDL, TG, BMI, Hb A1c.

**Dose-response: 0.** Dose-response relationship could not be detected in this article.

**Hamer M, Venuraju SM, Urbanova L, Lahiri A, and Steptoe A. Physical activity, sedentary time, and pericardial fat in healthy older adults. Obesity. 2012;20:2113–2117.**

**Design: 2.** Cross-Sectional

**Risk of Bias:** **-2**. **Selection Bias**: The participants included in the present analysis were generally healthier than the overall Whitehall II sample and demonstrated higher activity levels compared with similar aged British cohort. **Information bias**: The accelerometry device used in the present study could not distinguish between sitting and standing.

**Imprecision: 0**. Sedentary time was also associated with pericardial fat (β= 0.081, 95% CI, 0.022, 0.14), although associations for sedentary time did not remain significant, after adjustment for MVPA (β = 0.033, 95% CI, –0.031, 0.096).

**Indirectness:** **-1**. “Pericardial fat surrounds the coronary arteries and has been associated with cardiovascular risk factors and markers of subclinical atherosclerosis, independently of overall adiposity”

**Inconsistency: 0.** Heterogeneity not could be detected in this article.

**Magnitude of effect: 0.** Sedentary time was also associated with pericardial fat (β= 0.081, 95% CI, 0.022, 0.14), although associations for sedentary time did not remain significant, after adjustment for MVPA (β = 0.033, 95% CI, –0.031, 0.096).

**Confounding adjustment: +1.** Age, sex, registered time, BMI, HDL, LDL, blood pressure, glycated hemoglobin, smoking, statins, and MVPA.

**Dose-response: 0.** Dose-response relationship could not be detected in this article.

**Bankoski A, Harris TB, McClain JJ, Brychta RJ, Caserotti P, Chen KY, Berrigan D, Troiano RP, and Koster A. Sedentary Activity Associated With Metabolic Syndrome Independent of Physical Activity. Diabetes Care. 2011;34:497–503.**

**Design: 2.** Cross-Sectional

**Risk of Bias:** **0**. **Selection Bias**: no presence. **Misclassification bias**: no presence

**Imprecision: -1**. A higher percentage of time sedentary (Q1 vs Q2 - OR 1.58; 95% IC 1.03 - 2.24) and fewer sedentary breaks (OR 1.53; 95% CI 1.05 - 2.23) were associated with a signiﬁcantly greater likelihood of metabolic syndrome

**Indirectness:** **-1**. “People with metabolic syndrome have an increased risk to develop health problems such as heart disease, diabetes, and stroke”

**Inconsistency: 0.** Heterogeneity could not be detected in this article.

**Magnitude of effect: 0.** A higher percentage of time sedentary (Q1 vs Q2 - OR 1.58; 95% IC 1.03 - 2.24) and fewer sedentary breaks (OR 1.53; 95% CI 1.05 - 2.23) were associated with a signiﬁcantly greater likelihood of metabolic syndrome

**Confounding adjustment: +1.** Age, sex, ethnicity, education, alcohol intake, smoking, BMI, diabetes, heart disease and physical activity

**Dose-response: 0.** Dose-response relationship could not be detected in this article.

**Gomez-Cabello A, Pedreto-Chamizo R, Olivares PR, Hernández_Perera R, Rodríguez-Marroyo JA, Mata E, Aznar S, Villa JG, Espino-Torón L, Gusi N, González-Gross, Casajús JA, Ara I, and Vicente-Rodríguez G. Sitting time increases the overweight and obesity risk independently of walking time in elderly people from Spain. Maturitas. 2012;73(4):337–343.**

**Design: 2.** Cross-Sectional

**Risk of Bias:** **-1**. **Selection Bias**: Only independent no institutionalized elderly were included in the present study. **Information bias**: BMI is not a the best marker of body composition to determine metabolic risk.

**Imprecision: -1**. Sitting time increases the risk of overweight-obesity (1.42 OR; 95% CI 1.06 - 1.89) and overfat (1.4 OR; 95% CI 1.14 - 1.74) in women and the risk of central obesity (1.74 OR; 95% CI 1.212 – 2.488) in men, independetly of walking time..

**Indirectness:** **-1**. “Taking into account that overweight, obesity and central obesity are associated with an increased risk of certain pathologies among older adults, including hypertension, cardiovascular disease, diabetes, dyslipidemia, arthritis, some cancers [4] and also with an earlier morbidity and functional limitation”

**Inconsistency: 0.** Heterogeneity could not be detected in this article.

**Magnitude of effect: 0.** Sitting time increases the risk of overweight-obesity (1.42 OR; 95% CI 1.06 - 1.89) and overfat (1.4 OR; 95% CI 1.14 - 1.74) in women and the risk of central obesity (1.74 OR; 95% CI 1.212 - 2488) in men, independetly of walking time..

**Confounding adjustment: 0.** age and walking time

**Dose-response: 0.** Dose-response relationship could not be detected in this article..

**Gao X, Nelson ME, Tucker KL. Television viewing is associated with prevalence of metabolic syndrome in hispanic elders. Diabetes Care. 2007;30:694–700.**

**Design: 2.** Cross-Sectional

**Risk of Bias:** **0**. No presence. **Information bias**: No presence

**Imprecision: -1**. Each additional hour of television viewing was associated with a 19% greater likelihood of having the metabolic syndrome (odds ratio [OR] 1.19, 95% CI 1.1–1.3, P for trend 0.002). Subjects in the highest quartile of television watching had a risk for the metabolic syndrome 2.2 times (OR 2.2, 95% CI 1.1– 4.2) that for those in the lowest quartile

**Indirectness:** **-1**. “This syndrome is associated with diabetes, cardiovascular disease, and mortality.”

**Inconsistency: 0.** Heterogeneity could not be detected in this article.

**Magnitude of effect: +1.** Each additional hour of television viewing was associated with a 19% greater likelihood of having the metabolic syndrome (odds ratio [OR] 1.19, 95% CI 1.1–1.3, P for trend 0.002). Subjects in the highest quartile of television watching had a risk for the metabolic syndrome 2.2 times (OR 2.2, 95% CI 1.1– 4.2) that for those in the lowest quartile

**Confounding adjustment: +1.** age (years), sex, ethnicity, BMI (except for abdominal obesity, high waist-to-hip ratio, and high BMI), education (years), household arrangement (married/lives with spouse, unmarried/lives with others, and unmarried/lives alone), smoking (current, former, and never), and current alcohol use (heavy drinker: more than one drink/day for women or two drinks/day for men, moderate drinker: less than these, and nondrinker, based on 13.2 g alcohol/drink), total energy intake (MJ/day), saturated fat intake (% total energy), polyunsaturated fat intake (% total energy), trans fat intake (% total energy), fruit and vegetable intake (servings/day), and physical activity score (in quartiles), and daily living score

**Dose-response: +1.**  P for trend _0.002.

**Inoue S, Sugiyama T, Takamiya T, Oka K, Owen N, and Shimomitsu T. Television Viewing Time is Associated with Overweight/Obesity Among Older Adults, Independent of Meeting Physical Activity and Health Guidelines. J Epidemiol 2012;22(1):50-56**

**Design: 2.** Cross-Sectional

**Risk of Bias:** **-2**.**Selection Bias**: response rate: 66.9% **Information bias**: both the dependent and independent variables were measured by self-report

**Imprecision: -1**. As compared with the reference category (high TV/insufﬁcient MVPA), the adjusted ORs (95% CI) of overweight/obesity were 0.93 (0.65, 1.34) for high TV/sufﬁcient MVPA, 0.58 (0.37, 0.90) for low TV/insufﬁcient MVPA, and 0.67 (0.47, 0.97) for low TV/sufﬁcient MVPA.

**Indirectness:** **-1**. “might inﬂuence their cardiovascular health.”

**Inconsistency: 0.** Heterogeneity could not be detected in this article.

**Magnitude of effect: 0.** As compared with the reference category (high TV/insufﬁcient MVPA), the adjusted ORs (95% CI) of overweight/obesity were 0.93 (0.65, 1.34) for high TV/sufﬁcient MVPA, 0.58 (0.37, 0.90) for low TV/insufﬁcient MVPA, and 0.67 (0.47, 0.97) for low TV/sufﬁcient MVPA.

**Confounding adjustment: +1.** sex, age, education, employment status, city of residence, smoking, drinking, and physical functioning

**Dose-response: 0**. Dose-response relationship could not be detected in this article.

**Dogra S, Stathokostas L. Sedentary Behavior and Physical Activity Are Independent Predictors of Successful Aging in Middle-Aged and Older Adults. J Aging Res. 2012(2012);190654**

**Design: 2.** Cross-Sectional

**Risk of Bias:** **-1**.**Selection Bias**: No presence **Information bias**: both the dependent and independent variables were measured by self-report

**Imprecision: -1**. Least sedentary older (< 2 hours) were more likely to have overall successful aging (OR 1.43; 95% 1.23-1.67), physical successful aging (OR 2.44; 95% CI 1.64-3.65) and Sociological successful aging (OR 1.25; 95% CI 1.00-1.55)

**Indirectness:** 0. “Successful aging (SA); a term used to represent the physical, psychological, and social success with which adults age.”

**Inconsistency: 0.** Heterogeneity could not be detected in this article.

**Magnitude of effect: +1.** Least sedentary older (< 2 hours) were more likely to have overall successful aging (OR 1.43; 95% 1.23-1.67), physical successful aging (OR 2.44; 95% CI 1.64-3.65) and Sociological successful aging (OR 1.25; 95% CI 1.00-1.55).

**Confounding adjustment: +1.** age, marital status, income and physical activity

**Dose-response: 0**. A dose-response relationship was not tested. However, similarly, compared to sedentary older adults, moderately sedentary and least sedentary older adults were 38% (OR: 1.38; CI: 1.12–1.69) and 43% (OR: 1.43; CI: 1.23–1.67) more likely to be aging successfully overall.

**Pavey TG, Peeters GG, and Brown WJ. Sitting-time and 9-year all-cause mortality in older women. Br J Sports Med. 2012;0:1–5**

**Desing: 2.** Prospective Cohort

**Risk of Bias:** **-1**. **Selection Bias**: No presence **Information bias**: Although sitting measurement should be reliable and valid, self-reported instruments tends to misclassificate the information (non-differentially in this case).

**Imprecision: 0**. Sitting time 4 to <8 hours (HR 0.90; 95% CI 0.79-1.03), 8 to <11 (HR 1.21; 95% CI 1.01 – 1.44), >11 (HR 1.24; 95% CI 0.98 – 1.56). Trend HR 1.03 (1.01 – 1.05).

**Indirectness:** **0**. Mortality.

**Inconsistency: 0.** Heterogeneity was not measured found.

**Magnitude of effect: +1** Sitting time 4 to <8 hours (HR 0.90; 95% CI 0.79-1.03), 8 to <11 (HR 1.21; 95% CI 1.01 – 1.44), >11 (HR 1.24; 95% CI 0.98 – 1.56). Trend HR 1.03 (1.01 – 1.05). A signiﬁcant interaction ( p=0.02) was found between sitting-time and physical activity (PA), with increased mortality risk for prolonged sitting only among participants not meeting PA guidelines (HR for sitting =8 h/day: 1.31, 95% CI 1.07 to 1.61); HR for sitting =11 h/day: 1.47, CI 1.15 to 1.93).

**Confounding adjustment: +1.** age, education, marital status, area, smoking, alcohol consumption, BMI, physical activity, number of chronic conditions, self-reported health and assistance with daily tasks.

**Dose-response: +1.** Trend HR 1.03 (1.01 – 1.05)

**Gennuso KP, Gangnon RE, Matthews CE, Thraen-Borowski KM, and Colbert LH. Sedentary Behavior, Physical Activity, and Markers of Health in Older Adults. Med. Sci. Sports Exerc. 2013;45(8):1493.**

**Desing: 2.** Cross-Sectional

**Risk of Bias:** **0**.**Selection Bias**: No presence **Misclassification bias**: No presence

**Imprecision: -1**. Compared with sedentary, mortality HR were 0.91 (95% CI 0.76-1.10) in those who were newly sedentary, 0.86 (0.70-1.05) in formerly sedentary individuals, and 0.75 (0.62-0.90) in those who remained consistently non-sedentary. Less sedentary hour (first quartile) was protective for greater number of limitations in insufficient MVPA (0.48; 95% CI 0.29 – 0.79; P trend <001) and sufficient MVPA (0.83; 95% CI 0.43 – 1.62) when compared with higher quartile of sedentary hours.

**indirectness:** **-1**. “Cardiovascular disease, type 2 diabetes, cancer, mortality.”

**Inconsistency: 0.** Heterogeneity could not be detected in this article.

**Magnitude of effect: +1.** Compared with sedentary, mortality HR were 0.91 (95% CI 0.76-1.10) in those who were newly sedentary, 0.86 (0.70-1.05) in formerly sedentary individuals, and 0.75 (0.62-0.90) in those who remained consistently non-sedentary. Less sedentary hour (first quartile) was protective for greater number of limitations in insufficient MVPA (0.48; 95% CI 0.29 – 0.79; P trend <001) and sufficient MVPA (0.83; 95% CI 0.43 – 1.62) when compared with higher quartile of sedentary hours

**Confounding adjustment: +1.** sex, age, educational level, smoking, alcohol consumption, BMI, physical activity, chronic lung disease, ischemic heart disease, diabetes mellitus, osteomuscular disease, cancer, SF-36, limitations in mobility, and limitations in agility.

**Dose-response: +1**. Less sedentary hour (first quartile) was protective for greater number of limitations in insufficient MVPA (0.48; 95% CI 0.29 – 0.79; P trend <001). P trend <0.01 for weight, waist circumference and CRP.

**León-Muñoz LM, Martínez-Gómez D, Balboa-Castillo T, López-García E, Guallar-Castillón P, Rodríguez-Artalejo F. Continued Sedentariness, Change in Sitting Time, and Mortality in Older Adults. Med Sci Sports Exerc. 2013;45(8):1501-1507.**

**Desing: 2.** Prospective Cohort

**Risk of Bias:** **-1**. **Selection Bias**: No presence **Information bias**: Although sitting measurement should be reliable and valid, self-reported instruments tends to misclassificate the information (non-differentially in this case).

**Imprecision: 0**. Compared with sedentary, mortality HR were 0.91 (95% CI 0.76-1.10) in those who were newly sedentary, 0.86 (0.70-1.05) in formerly sedentary individuals, and 0.75 (0.62-0.90) in those who remained consistently non-sedentary

**Indirectness:** **0**. Mortality.

**Inconsistency: 0.** Heterogeneity was not measured found.

**Magnitude of effect: +1** Compared with sedentary, mortality HR were 0.91 (95% CI 0.76-1.10) in those who were newly sedentary, 0.86 (0.70-1.05) in formerly sedentary individuals, and 0.75 (0.62-0.90) in those who remained consistently non-sedentary

**Confounding adjustment: +1.** sex, age, educational level, smoking, alcohol consumption, BMI, physical activity, chronic lung disease, ischemic heart disease, diabetes mellitus, osteomuscular disease, cancer, SF-36, limitations in mobility, and limitations in agility.

**Dose-response: +1.** The average ST between 2001 and 2003 showed an inverse dose–response relationship (P for trend G0.001) with all-cause mortality from 2003 through 2011

**Verghese J, Lipton RB, Katz MJ, Hall CB, Derby CA, Kuslansky G, mabrose AF, Sliwinski M, and Buschke H. Leisure Activities and the Risk of Dementia in the Elderly. N Engl J Med. 2003;348(25):2508-2516.**

**Design: 2.** Prospective Cohort

**Risk of Bias:** **-2**. **Selection Bias**: volunteers who resided in the community; whites and subjects older than 75 years of age were overrepresented, as compared with the general population of those over 65 years of age **Information bias**: Although leisure activities measurement should be reliable and valid, self-reported instruments tends to misclassificate the information (non-differentially in this case).

**Imprecision: 0**. Individuals that frequent are engaged in playing board games (HR 0.26; 95% CI 0.17-0.57), reading (HR 0.65; 95% CI 0.43-0.97), and playing musical instrument (HR 0.31; 95% CI 0.11-0.90) was less likely to develop dementia when compared with rare group.

**Indirectness:** **0**. Dementia.

**Inconsistency: 0.** Heterogeneity was not measured found.

**Magnitude of effect: +1** Individuals that frequent are engaged in playing board games (HR 0.26; 95% CI 0.17-0.57), reading (HR 0.65; 95% CI 0.43-0.97), and playing musical instrument (HR 0.31; 95% CI 0.11-0.90) was less likely to develop dementia when compared with rare group.

**Confounding adjustment: +1.** age, sex, educational level, presence or absence of medical illnesses, score on the Blessed Information–Memory–Concentration test, and participation or nonparticipation in other leisure activities.

**Dose-response: 0.** A dose-response relationship was not tested

**Geda YE, Topazian HM, Roberts LA, Roberts RO, Knopman DS, Pankratz VS, Christianson TJ, Boeve BF, Tangalos EG, Ivnik RJ, Petersen RC. Engaging in cognitive activities, aging, and mild cognitive impairment: a population based study. J Neuropsychiatry Clin Neurosci. 2011;23(2):149-154.**

**Desing: 2.** Cross-Sectional

**Risk of Bias:** **-1**.**Selection Bias**: No presence **Information bias**: Although television time measurement should be reliable and valid, self-reported instruments tends to misclassificate the information (non-differentially in this case).

**Imprecision: 0**. Reading books (OR 0.67; 95% CI 0.49 –0.94), playing games (OR 0.65; 95% CI 0.47– 0.90), craft activities (OR 0.66; 95% CI 0.47– 0.93), computer activities (OR 0.50; 95% CI 0.36 –0.71), and watching television (OR 0.48; 95% CI 0.27– 0.86) were signiﬁcantly associated with decreased odds of having MCI

**Indirectness:** **-1**. “Mild cognitive impairment (MCI) is the intermediate stage between the cognitive changes of nor- mal aging and those of dementia.”

**Inconsistency: 0.** Heterogeneity could not be detected in this article.

**Magnitude of effect: +1.** Reading books (OR 0.67; 95% CI 0.49 –0.94), playing games (OR 0.65; 95% CI 0.47– 0.90), craft activities (OR 0.66; 95% CI 0.47– 0.93), computer activities (OR 0.50; 95% CI 0.36 –0.71), and watching television (OR 0.48; 95% CI 0.27– 0.86) were signiﬁcantly associated with decreased odds of having MCI

**Confounding adjustment: +1.** age, sex, education, depression, medical comorbidity, and physical exercise

**Dose-response: 0**. A dose-response relationship was not tested
